# Recognizing protein–protein interfaces with empirical potentials and reduced amino acid alphabets

**DOI:** 10.1186/1471-2105-8-270

**Published:** 2007-07-27

**Authors:** Guillaume Launay, Raul Mendez, Shoshana Wodak, Thomas Simonson

**Affiliations:** 1 Laboratoire de Biochimie (UMR CNRS 7654), Department of Biology, Ecole Polytechnique, 91128, Palaiseau, France; 2 Service de Conformation de Macromolécules Biologiques et Bioinformatique, Centre de Biologie Structurale et Bioinformatique, Université Libre de Bruxelles, Belgium; 3 Structural Biology Program, Hospital for Sick Children, Toronto, Canada

## Abstract

**Background:**

In structural genomics, an important goal is the detection and classification of protein–protein interactions, given the structures of the interacting partners. We have developed empirical energy functions to identify native structures of protein–protein complexes among sets of decoy structures. To understand the role of amino acid diversity, we parameterized a series of functions, using a hierarchy of amino acid alphabets of increasing complexity, with 2, 3, 4, 6, and 20 amino acid groups. Compared to previous work, we used the simplest possible functional form, with residue–residue interactions and a stepwise distance-dependence. We used increased computational ressources, however, constructing 290,000 decoys for 219 protein–protein complexes, with a realistic docking protocol where the protein partners are flexible and interact through a molecular mechanics energy function. The energy parameters were optimized to correctly assign as many native complexes as possible. To resolve the multiple minimum problem in parameter space, over 64000 starting parameter guesses were tried for each energy function. The optimized functions were tested by cross validation on subsets of our native and decoy structures, by blind tests on series of native and decoy structures available on the Web, and on models for 13 complexes submitted to the CAPRI structure prediction experiment.

**Results:**

Performance is similar to several other statistical potentials of the same complexity. For example, the CAPRI target structure is correctly ranked ahead of 90% of its decoys in 6 cases out of 13. The hierarchy of amino acid alphabets leads to a coherent hierarchy of energy functions, with qualitatively similar parameters for similar amino acid types at all levels. Most remarkably, the performance with six amino acid classes is equivalent to that of the most detailed, 20-class energy function.

**Conclusion:**

This suggests that six carefully chosen amino acid classes are sufficient to encode specificity in protein–protein interactions, and provide a starting point to develop more complicated energy functions.

## Background

An important goal of modern genomics is to identify interacting proteins and characterize the structure and function of the corresponding complexes [1-3]. There are many complexes in even a simple organism; for example several hundred have been identified in yeast [4]. Experimental structure determination for all of them is impractical, although determining a representative set should eventually be possible [5]. Therefore, methods for structure prediction are useful [6]. The most tractable situation occurs when the structures of the two partners are known separately. Indeed, in many (though not all) protein–protein complexes, the three-dimensional structure of each partner is very close to its structure when alone. In this case, the prediction amounts to positioning one protein with respect to the other. The problem can be divided further into two parts: generating a number of reasonable, putative, complex structures (the "docking" problem), and identifying the correct one [7-9]. The second problem, recognizing the biologically correct complex among a possibly-large set of candidate structures, is referred to as the "scoring" or "interface recognition" problem.

In this work, we develop a series of energy functions for interface recognition. To handle many proteins on a genomic scale, the computational model should be simple and efficient [10]. Therefore, following many previous workers, we take a coarse-grained view of the protein structures, with interactions described at the amino acid level, and parameterize the model empirically. Protein quaternary structure analysis is an active field [11-17], and several protein–protein interaction potentials have already been developed. Information on protein structures has been used, as well as sequence conservation and covariance between interacting partners [7,18-23]. Sequence conservation and three-dimensional structures give different information and should be combined to provide the maximum predictive power. Here, we take the view that sequence conservation can be used in a separate step to filter possible models, and we parameterize our model using structural information only.

We pursue two main goals. First, with increased computing power, we can employ larger numbers of structures and more realistic decoys than in previous work, which should lead to improved parameterization. Second and more importantly, we consider a series of models of increasing complexity. The simplest one distinguishes just two types of amino acids: hydrophobic and polar. The most complex one distinguishes all twenty amino acid types. The intermediate ones distinguish three, four, and six amino acid types. By deriving a hierarchy of energy functions, we can determine what model complexity is needed to solve the interface recognition problem.

A third goal is to help clarify an important biological problem: what amount of chemical diversity is needed to construct specific protein–protein interfaces. Indeed, the structures and interactions of natural proteins are (almost always) encoded in their amino acid sequences. However, chemically similar amino acids in a protein can often be interchanged without altering noticeably the structure, and protein engineers have already reproduced naturally occuring protein folds using reduced sets of amino acids [24]. Very early stages in evolution may have made use of such reduced sets of amino acids.

The energy function tested in this work relies on pairwise amino acid potentials with the simplest possible, stepwise, distance-dependence: the interaction energy between two amino acids is zero above a certain distance threshold and constant below it. Potentials with a more complex distance-dependence are known to give superior results [21,25,26]. Nevertheless, we focused on the present, stepwise potentials in order to analyse the effect of reduced amino acid alphabets in a simple context.

To parameterize the energy functions, we use an optimization method introduced earlier for fold recognition [20]. The goal is to obtain interresidue interaction parameters that assign a low energy to the correct, native complex structure, and a higher energy to alternate, incorrect structures. A dataset of 219 protein–protein complexes of known structure are used to train and test the models. For each one, about 1300 decoy structures are generated using a realistic docking procedure, in which the protein partners are flexible and interact through a molecular mechanics energy function. The decoy sets will be described in detail elsewhere. Half of the structures form an "Optimization Set"; the others form a "Test Set". As an additional restraint on the energy function, we include in the optimization criterion its performance for fold recognition, using an analogous dataset of 800 monomeric protein structures with associated decoys.

The energy functions are subjected to a series of blind tests. These involve our own Test Set of native and decoy structures, sets of native and decoy structures made available by Sternberg, Vakser and coworkers through the World Wide Web [27,28], and 13 target structures submitted to the CAPRI experiment for protein–protein complex structure prediction (rounds 2–5). Results are comparable to two other, recent, empirical models due to Bastolla, Lu et al [19,20]. For example, the CAPRI target structure is correctly ranked ahead of 90% of its decoys in 6 cases out of 13. However, our hierarchical approach yields valuable additional insights. Thus, the hierarchy of amino acid alphabets leads to a coherent hierarchy of energy functions, with qualitatively similar parameters for similar amino acid types at all levels. Remarkably, the performance with six amino acid classes is equivalent to that of the most detailed. 20-class energy function, and the performance with four classes is only slightly worse. This indicates that six or even four carefully chosen amino acid classes are sufficient to encode the true complexity of the residue–residue interaction model. It suggests that six amino acid types are also sufficient to provide interface specificity for many protein–protein complexes.

## Results

We first describe briefly the optimization of energy functions for fold recognition of monomeric proteins [29] and their behavior with amino acid alphabets of increasing complexity. Then we consider the recognition of protein–protein interfaces. Finally, we describe the application to a series of blind tests. In Supplementary Material, we analyze the convergence and robustness of the parameter optimization procedure (see additional file 1: Supp1.ps).

### Fold recognition for monomeric structures

**Figure 1:**
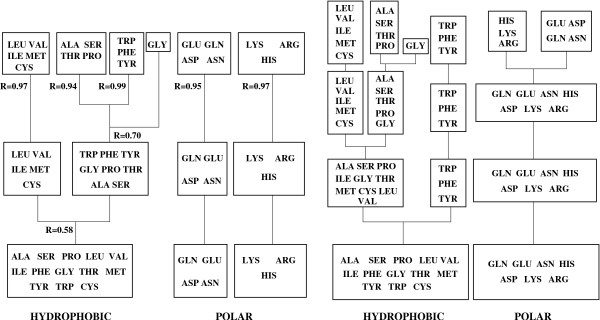
**Hierarchical Amino Acid Classification Tree**. Hierarchical clustering of amino acid types, using the Blosum50 similarity matrix (right) or the optimized, 20-class energy matrix (left). The Pearson Correlation coefficent of each cluster is given for the lefthand tree.

The energy function is a sum over pairs of residues less than 4.5 Å apart. Each pair of residue types has its own interaction parameter. Several parameter optimizations were conducted, using the amino acid alphabets in Fig. 1. which contain 2, 3, 4, 6, and 20 amino acid types, respectively. We considered 810 native protein structures, along with sets of decoys constructed by threading each sequence onto unrelated structures. The structures and their decoys were split into an Optimization set (615 structures) and a Test set (200 structures). The energy parameters were chosen to maximize the Q¯
 MathType@MTEF@5@5@+=feaafiart1ev1aaatCvAUfKttLearuWrP9MDH5MBPbIqV92AaeXatLxBI9gBaebbnrfifHhDYfgasaacH8akY=wiFfYdH8Gipec8Eeeu0xXdbba9frFj0=OqFfea0dXdd9vqai=hGuQ8kuc9pgc9s8qqaq=dirpe0xb9q8qiLsFr0=vr0=vr0dc8meaabaqaciaacaGaaeqabaqabeGadaaakeaacuWGrbqugaqeaaaa@2DEF@ parameter (Eq. 7), summed over the proteins of the Optimization set. The quality of each energy function can be measured by the final, optimized Q¯
 MathType@MTEF@5@5@+=feaafiart1ev1aaatCvAUfKttLearuWrP9MDH5MBPbIqV92AaeXatLxBI9gBaebbnrfifHhDYfgasaacH8akY=wiFfYdH8Gipec8Eeeu0xXdbba9frFj0=OqFfea0dXdd9vqai=hGuQ8kuc9pgc9s8qqaq=dirpe0xb9q8qiLsFr0=vr0=vr0dc8meaabaqaciaacaGaaeqabaqabeGadaaakeaacuWGrbqugaqeaaaa@2DEF@ value, which ranges from 0 to 100% and reflects the ability of the energy function to group near-native structures in the low-energy range. A second quality measure is the "discrimination percentage" *D*: the percentage of native structures in the Optimization set that are successfully predicted to be of lower energy than all their decoys. The quality measures are obtained by cross validation; *i.e*., we compute Q¯
 MathType@MTEF@5@5@+=feaafiart1ev1aaatCvAUfKttLearuWrP9MDH5MBPbIqV92AaeXatLxBI9gBaebbnrfifHhDYfgasaacH8akY=wiFfYdH8Gipec8Eeeu0xXdbba9frFj0=OqFfea0dXdd9vqai=hGuQ8kuc9pgc9s8qqaq=dirpe0xb9q8qiLsFr0=vr0=vr0dc8meaabaqaciaacaGaaeqabaqabeGadaaakeaacuWGrbqugaqeaaaa@2DEF@ and *D *for the structures of the Test set, which are not used for parameter optimization. The quality of the predictions are summarized in Table 1.

**Table 1:** Fold recognition: performance of the optimized energy functions on the Optimization and Test sets

Number of amino acid groups	Optimization Set		Test Set	
	Q¯ MathType@MTEF@5@5@+=feaafiart1ev1aaatCvAUfKttLearuWrP9MDH5MBPbIqV92AaeXatLxBI9gBaebbnrfifHhDYfgasaacH8akY=wiFfYdH8Gipec8Eeeu0xXdbba9frFj0=OqFfea0dXdd9vqai=hGuQ8kuc9pgc9s8qqaq=dirpe0xb9q8qiLsFr0=vr0=vr0dc8meaabaqaciaacaGaaeqabaqabeGadaaakeaacuWGrbqugaqeaaaa@2DEF@(%)	*D *(%)	Q¯ MathType@MTEF@5@5@+=feaafiart1ev1aaatCvAUfKttLearuWrP9MDH5MBPbIqV92AaeXatLxBI9gBaebbnrfifHhDYfgasaacH8akY=wiFfYdH8Gipec8Eeeu0xXdbba9frFj0=OqFfea0dXdd9vqai=hGuQ8kuc9pgc9s8qqaq=dirpe0xb9q8qiLsFr0=vr0=vr0dc8meaabaqaciaacaGaaeqabaqabeGadaaakeaacuWGrbqugaqeaaaa@2DEF@(%)	*D*(%)
2	74.3	69.8	74.4	70.0
3	76.4	72.2	75.3	72.0
4	87.5	84.4	87.4	84.6
6	88.3	86.7	91.9	89.4
20	94.4	91.9	91.3	88.4

The simplest amino acid alphabet is a binary one, with an approximate separation into hydrophobic and hydrophilic amino acids (Fig. 1). The corresponding optimal parameters are given in Table 2. They assign favorable energies to intraclass contacts and an unfavorable energy to interclass (hydrophobic-hydrophilic) contacts. This very simple model already gives a respectable discrimination, (*D *= 70%, Q¯
 MathType@MTEF@5@5@+=feaafiart1ev1aaatCvAUfKttLearuWrP9MDH5MBPbIqV92AaeXatLxBI9gBaebbnrfifHhDYfgasaacH8akY=wiFfYdH8Gipec8Eeeu0xXdbba9frFj0=OqFfea0dXdd9vqai=hGuQ8kuc9pgc9s8qqaq=dirpe0xb9q8qiLsFr0=vr0=vr0dc8meaabaqaciaacaGaaeqabaqabeGadaaakeaacuWGrbqugaqeaaaa@2DEF@ = 74%), partly due to the simplicity of our monomeric decoy set, obtained by a rudimentary threading procedure (see Materials and Methods). The three residue alphabet puts the aromatic residues in a class of their own. The corresponding energy parameters (Table 2) are qualitatively consistent with the binary ones. The discriminating power is slightly increased (*D *= 72%, Q¯
 MathType@MTEF@5@5@+=feaafiart1ev1aaatCvAUfKttLearuWrP9MDH5MBPbIqV92AaeXatLxBI9gBaebbnrfifHhDYfgasaacH8akY=wiFfYdH8Gipec8Eeeu0xXdbba9frFj0=OqFfea0dXdd9vqai=hGuQ8kuc9pgc9s8qqaq=dirpe0xb9q8qiLsFr0=vr0=vr0dc8meaabaqaciaacaGaaeqabaqabeGadaaakeaacuWGrbqugaqeaaaa@2DEF@ = 75%).

**Table 2:** Two- and three-class energy parameters for fold recognition and dimer interface recognition. Best parameters (kcal/mol) for monomeric fold recognition (upper right) and dimer interface recognition (lower left).

		H	P	**Monomeric**	
		-8.5	9.0	H = {ALSVTPIGMCFYW}	
H	-9.0		-3.5	P = {EKRHDNQ}	
P	9.8	-7.1			
**Dimeric**	H	P			

		H_*a*_	H_*b*_	P	**Monomeric**

		-4.7	-9.6	6.4	H_*a *_= {ALSVTPIGMC}
H_*a*_	-3.8		-11.4	1.5	H_*b *_= {FYW}
H_*b*_	-8.4	-14.3		-1.9	P = {EKRHDNQ}
P	4.8	2.4	-1.5		
**Dimeric**	H_*a*_	H_*b*_	P		

With four and six classes, the discrimination *D *increases markedly, to 85% and 89%, respectively (Table 1). The four-class alphabet puts L, V, I, M and C into a separate group. The parameter values (Table 3) remain consistent with the simpler alphabets. For example, aromatic–aromatic interactions remain very favorable, while polar–polar interactions remain unfavorable, except for those between the two classes {EDNQ} and {KRH}.

**Table 3:** Four- and six-class energy parameters for fold recognition and dimer interface recognition. Best four- and six-class energy parameters (kcal/mol) for monomeric proteins (upper right) and dimer interface recognition (lower left)

			H_*α*_	H_*β*_	H_*γ*_	P	**Monomeric**	
			-6.96	1.81	-8.85	1.94	H_*α *_= {LVIMC}	
	H_*α*_	-5.70		-0.59	0.15	1.56	H_*β *_= {AGSTP}	
	H_*β*_	-0.60	-0.02		-6.41	1.53	H_*γ *_= {FYW}	
	H_*γ*_	-3.68	-0.61	-7.08		-1.30	P = {EDNQKRH}	
	P	1.75	0.04	0.63	1.21			

	**Dimeric**	H_*α*_	H_*β*'_	H_*γ*_	P			

		H_*α*_	H_*β'*_	H_*γ*_	*P*_*α*_	*P*_*β*_	G	**Monomeric**

		-8.30	0.30	-5.11	3.77	2.62	0.43	H_*α *_= {LVIMC}
H_*α*_	-2.06		-1.35	1.49	0.42	1.30	2.54	H_*β' *_= {ASTP}
H_*β'*_	0.09	-0.02		-7.79	1.01	0.79	-0.85	H_*γ *_= {FYW}
H_*γ*_	-1.73	-0.45	-0.65		1.61	-3.30	1.00	P_*α *_= {EDNQ}
P_*α*_	0.76	0.13	0.36	0.52		1.57	-0.38	P_*β *_= {KRH}
P_*β*_	0.51	-0.24	-0.25	-0.30	0.53		-0.08	G
G	-0.17	0.12	-0.55	0.04	0.19	0.01		

**Dimeric**	H_*α*_	H_*β'*_	H_*γ*_	P_*α*_	P_*β*_	G		

The most detailed energy function, with all twenty amino acid types, is subject to overoptimization, or overtraining: performance on the Test set is 3–4% below that on the Optimization set (Table 1). In fact, the performance on the Test set is slightly inferior (by 1%) to that of the best six-class energy function. For the simpler alphabets (2–6 types), the performance on the Test set was as good, or slightly better than on the Optimization set.

**Figure 2:**
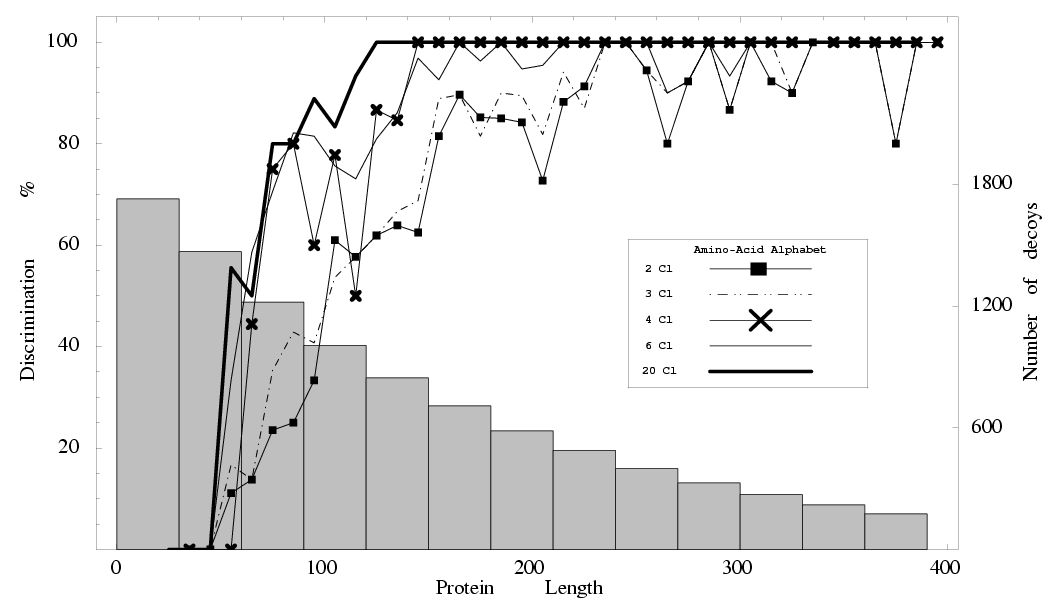
**Discrimination according to Protein Size**. Discrimination power of the different amino acid alphabets for fold recognition as a function of protein length (number of amino acids). The corresponding energy functions are those derived for fold recognition, using the Monomeric Optimization Set. The mean number of decoys is shown vs. protein length (grey bars; righthand graduations).

Fig. 2 shows the discrimination *D *for different protein size ranges, for all alphabets. For proteins of more than 320 amino acids, discrimination is 100% with either four, six, or twenty residue classes. With two and three amino acid classes, discrimination reaches 100% when the protein size reaches 250 amino acids, except for a few large proteins. The lower discrimination for small proteins is partly due to their higher number of decoys (also shown).

The amino acid groupings used here (Fig. 1) were derived from the Blosum50 similarity matrix by Levy et al [30]. Residues with similar physicochemical properties are clustered together. The parameters show a good consistency with the hierarchy of amino acid types, as discussed above. When an amino acid group is subdivided, the parameters corresponding to the subgroups are usually in qualitative agreement with the parent group. This is not due to bias in the optimization procedure, since the best parameters at each level were usually obtained from a random scan of parameter space (see Methods). Another six-class grouping ({AVLIMC}, {FWYH}, {STNQ}, {KR}, {DE}, {GP}), proposed by Shakhnovich and coworkers [31], gave significantly poorer results, with a *D *of 71%.

### Recognizing protein–protein interfaces

#### Characterizing the structures

**Figure 3:**
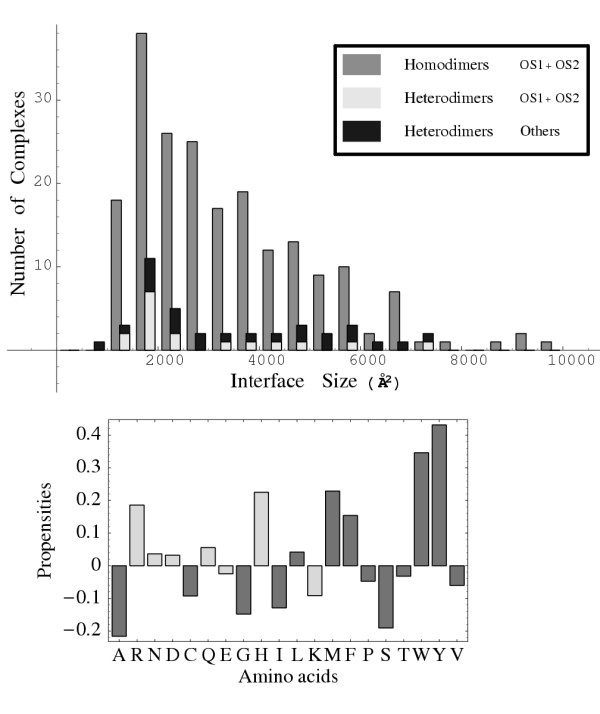
**Characterizing the dimeric protein complexes **. Upper panel: the distribution of interface sizes among the 219 complexes. Lower panel: the propensity *P*_X _of each amino acid type *X *to be found in the interface (Eq. 1). Positive (negative) values correspond to types that are overrepresented (underrepresented) in the interfaces, compared to their abundance in the SwissProt data bank. The two shades of grey correspond to the binary amino acid classification.

To parameterize and test the energy functions, we used 219 protein–protein complex structures, including 195 homodimers and 24 heterodimers. We constructed about 1300 decoys each (for a total of about 290,000 decoys). The decoys were constructed by a realistic (and computationally expensive) docking procedure, with flexible proteins interacting through a molecular mechanics energy function. Some of the native structures' properties are summarized in Fig. 3. The distribution of interface sizes has a peak at about 1800 Å^2^, with a broad tail extending up to about 6500 Å^2^. The propensities *P*_*X *_of each amino acid type *X *to be found at the interface are also shown. *P*_*X *_is defined as

PX=log⁡fXint⁡/fXSWP,
 MathType@MTEF@5@5@+=feaafiart1ev1aaatCvAUfKttLearuWrP9MDH5MBPbIqV92AaeXatLxBI9gBaebbnrfifHhDYfgasaacH8akY=wiFfYdH8Gipec8Eeeu0xXdbba9frFj0=OqFfea0dXdd9vqai=hGuQ8kuc9pgc9s8qqaq=dirpe0xb9q8qiLsFr0=vr0=vr0dc8meaabaqaciaacaGaaeqabaqabeGadaaakeaacqWGqbaudaWgaaWcbaGaemiwaGfabeaakiabg2da9iGbcYgaSjabc+gaVjabcEgaNjabdAgaMnaaDaaaleaacqWGybawaeaacyGGPbqAcqGGUbGBcqGG0baDaaGccqGGVaWlcqWGMbGzdaqhaaWcbaGaemiwaGfabaGaee4uamLaee4vaCLaeeiuaafaaOGaeiilaWcaaa@4371@

where fXint⁡
 MathType@MTEF@5@5@+=feaafiart1ev1aaatCvAUfKttLearuWrP9MDH5MBPbIqV92AaeXatLxBI9gBaebbnrfifHhDYfgasaacH8akY=wiFfYdH8Gipec8Eeeu0xXdbba9frFj0=OqFfea0dXdd9vqai=hGuQ8kuc9pgc9s8qqaq=dirpe0xb9q8qiLsFr0=vr0=vr0dc8meaabaqaciaacaGaaeqabaqabeGadaaakeaacqWGMbGzdaqhaaWcbaGaemiwaGfabaGagiyAaKMaeiOBa4MaeiiDaqhaaaaa@3397@ and fXSWP
 MathType@MTEF@5@5@+=feaafiart1ev1aaatCvAUfKttLearuWrP9MDH5MBPbIqV92AaeXatLxBI9gBaebbnrfifHhDYfgasaacH8akY=wiFfYdH8Gipec8Eeeu0xXdbba9frFj0=OqFfea0dXdd9vqai=hGuQ8kuc9pgc9s8qqaq=dirpe0xb9q8qiLsFr0=vr0=vr0dc8meaabaqaciaacaGaaeqabaqabeGadaaakeaacqWGMbGzdaqhaaWcbaGaemiwaGfabaGaee4uamLaee4vaCLaeeiuaafaaaaa@32F0@ are the proportion of amino acid type *X *found in the protein–protein interfaces and in the SwissProt data base, respectively [13]. A positive propensity *P*_*X *_means that the proportion of *X *in the interfaces is higher than the overall proportion of *X *in SwissProt. Hydrophobic types, especially Met, Trp, Tyr and Phe are overrepresented, compared to their natural abundance in proteins. Among the polar amino acids, Arg and His are overrepresented, and Lys is underrepresented. Homodimeric interfaces (the majority of our data set) tend to be larger and more hydrophobic than heterodimers. Nevertheless, the trends in Fig. 3 agree with recent surveys of protein–protein interfaces [12,13].

**Figure 4:**
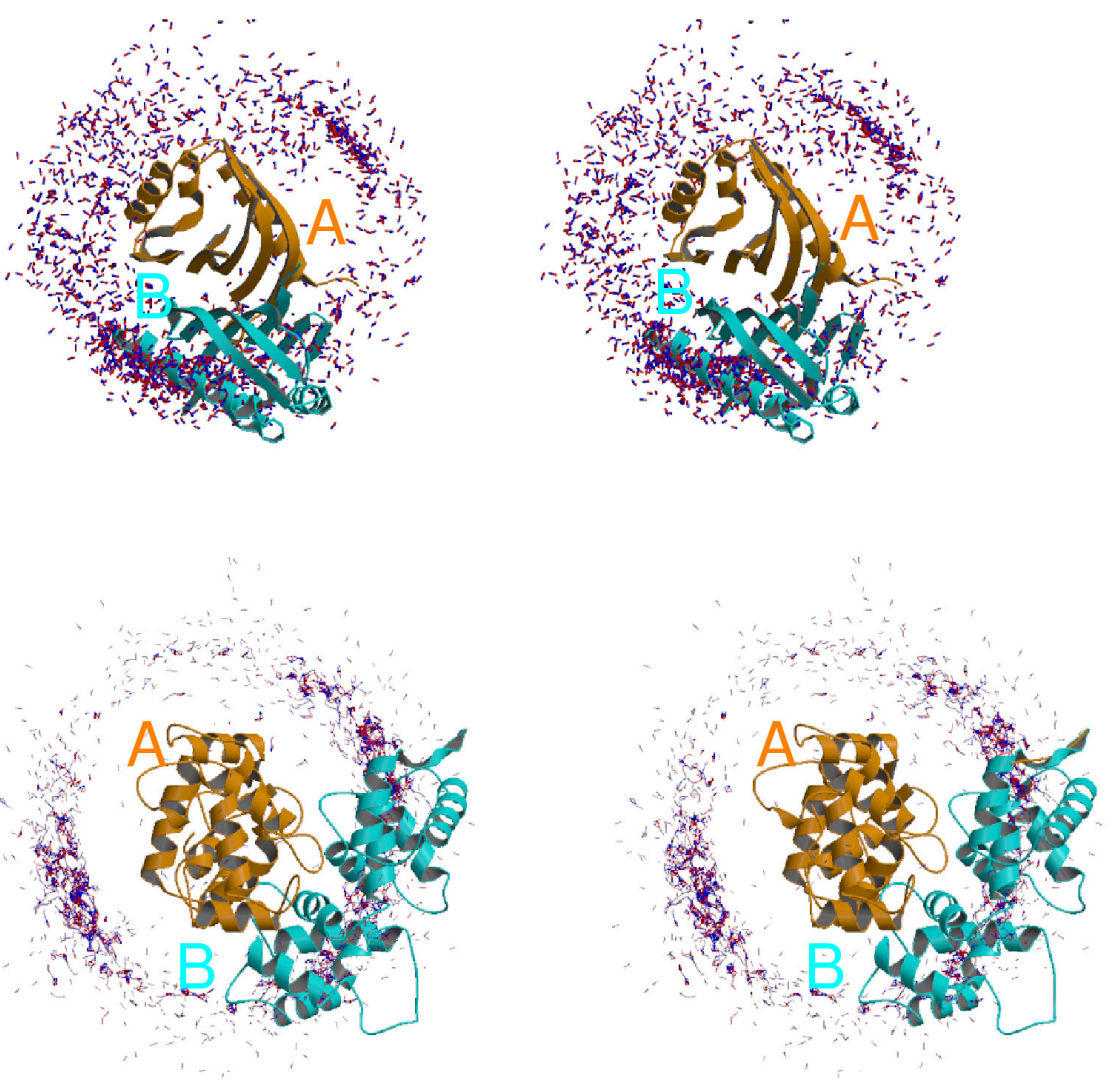
**Coverage of the surface of the receptors**. Two examples of decoy structure series, along with their native structures: 1ARO (top) and 1BJF (bottom). In each case, the native complex is shown. One partner (the 'A' receptor) is arbitrarily taken as a reference (orange ribbon). The second partner (the 'B' ligand) is shown as a cyan tube. The decoys corresponding to B are schematized by sticks; constructed from the center of mass and an arbitrary atom in the decoy structure.

**Figure 5:**
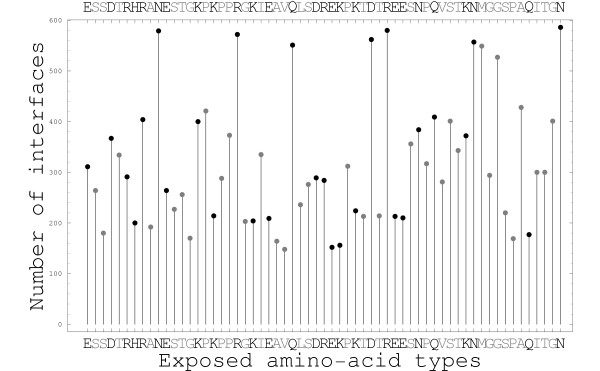
**Coverage of the surface of the receptors**. Surface coverage of one partner by the other for the (homodimeric) 1AA7 decoy series (total of 1507 decoys). For each surface amino acid in one partner, we show the number of decoys where it is part of the interface (ie, buried by the other partner). Polar/nonpolar amino acids are in black/grey. We see that all the amino acids at the protein surface participate in the interfaces of many decoys.

To illustrate our decoy sets, Fig. 4 shows two native structures (PDB codes 1ARO and 1BJF) along with a simplified representation of their decoy series. The series contain 1573 and 1652 decoys each. Decoys are produced by fixing one or the other of the partners, and positioning the other using a flexible docking procedure (see Methods). In Fig. 4. one partner ("A") is arbitrarily taken as a reference, and the distribution of the other ("B") around it is shown (one structure per decoy). Partner B is seen to be widely distributed around A, with a wide variety of orientations. By construction, each decoy has a native-like interface size and a limited distortion of the internal structure of both A and B. The energy distributions for these same series of structures are discussed in the next section. The decoy series for a third, typical, dimer is illustrated in Fig. 5 in a different way. For each amino acid of one member of the dimer, Fig. 5 shows the number of decoys where it participates in the protein–protein interface. We see, again, that the surface coverage of one partner by the other in the decoy sets is rather thorough.

#### Recognition power with different amino acid alphabets

The energy functions for interface recognition were optimized using a combination of monomeric and dimeric structures (see Methods). We considered two Optimization sets, containing respectively 110 and 109 dimeric structures and their associated decoys, along with 110 monomeric structures and their decoys. We denote them OS1 and OS2. The dimeric structures not belonging to OS1 form the Test Set 1; similarly for OS2. The amino acid subdivisions into classes are the same as for the monomeric energy functions above. The prediction results are summarized in Table 4. The optimized energy parameters are in Tables 2, 3 and in Supplementary Material (for the 20 class function: see additional file 2: Supp2.ps). To measure performance, we employ *D*_*int*_, the percentage of native interfaces ranked above all their decoys, and Q¯int
 MathType@MTEF@5@5@+=feaafiart1ev1aaatCvAUfKttLearuWrP9MDH5MBPbIqV92AaeXatLxBI9gBaebbnrfifHhDYfgasaacH8akY=wiFfYdH8Gipec8Eeeu0xXdbba9frFj0=OqFfea0dXdd9vqai=hGuQ8kuc9pgc9s8qqaq=dirpe0xb9q8qiLsFr0=vr0=vr0dc8meaabaqaciaacaGaaeqabaqabeGadaaakeaacuWGrbqugaqeamaaBaaaleaacqWGPbqAcqWGUbGBcqWG0baDaeqaaaaa@324C@, the average of Q¯
 MathType@MTEF@5@5@+=feaafiart1ev1aaatCvAUfKttLearuWrP9MDH5MBPbIqV92AaeXatLxBI9gBaebbnrfifHhDYfgasaacH8akY=wiFfYdH8Gipec8Eeeu0xXdbba9frFj0=OqFfea0dXdd9vqai=hGuQ8kuc9pgc9s8qqaq=dirpe0xb9q8qiLsFr0=vr0=vr0dc8meaabaqaciaacaGaaeqabaqabeGadaaakeaacuWGrbqugaqeaaaa@2DEF@ over all the protein–protein complexes. *D*_*int *_and Q¯
 MathType@MTEF@5@5@+=feaafiart1ev1aaatCvAUfKttLearuWrP9MDH5MBPbIqV92AaeXatLxBI9gBaebbnrfifHhDYfgasaacH8akY=wiFfYdH8Gipec8Eeeu0xXdbba9frFj0=OqFfea0dXdd9vqai=hGuQ8kuc9pgc9s8qqaq=dirpe0xb9q8qiLsFr0=vr0=vr0dc8meaabaqaciaacaGaaeqabaqabeGadaaakeaacuWGrbqugaqeaaaa@2DEF@ are exactly analogous to *D *and Q¯
 MathType@MTEF@5@5@+=feaafiart1ev1aaatCvAUfKttLearuWrP9MDH5MBPbIqV92AaeXatLxBI9gBaebbnrfifHhDYfgasaacH8akY=wiFfYdH8Gipec8Eeeu0xXdbba9frFj0=OqFfea0dXdd9vqai=hGuQ8kuc9pgc9s8qqaq=dirpe0xb9q8qiLsFr0=vr0=vr0dc8meaabaqaciaacaGaaeqabaqabeGadaaakeaacuWGrbqugaqeaaaa@2DEF@ (Eq. 7), but they only include the interface recognition statistics; fold recognition statistics are not included (whereas they are taken into account during the optimization).

**Table 4:** Dimer interface recognition: performance of the energy functions

Number of amino acid groups	Optimization Set 1^*a*^				Optimization Set 2^*b*^			
	Opt.^*c*^	Test.^*d*^			Opt.^*c*^	Test^*d*^		
	Q¯ MathType@MTEF@5@5@+=feaafiart1ev1aaatCvAUfKttLearuWrP9MDH5MBPbIqV92AaeXatLxBI9gBaebbnrfifHhDYfgasaacH8akY=wiFfYdH8Gipec8Eeeu0xXdbba9frFj0=OqFfea0dXdd9vqai=hGuQ8kuc9pgc9s8qqaq=dirpe0xb9q8qiLsFr0=vr0=vr0dc8meaabaqaciaacaGaaeqabaqabeGadaaakeaacuWGrbqugaqeaaaa@2DEF@	Q¯ MathType@MTEF@5@5@+=feaafiart1ev1aaatCvAUfKttLearuWrP9MDH5MBPbIqV92AaeXatLxBI9gBaebbnrfifHhDYfgasaacH8akY=wiFfYdH8Gipec8Eeeu0xXdbba9frFj0=OqFfea0dXdd9vqai=hGuQ8kuc9pgc9s8qqaq=dirpe0xb9q8qiLsFr0=vr0=vr0dc8meaabaqaciaacaGaaeqabaqabeGadaaakeaacuWGrbqugaqeaaaa@2DEF@	Q¯int MathType@MTEF@5@5@+=feaafiart1ev1aaatCvAUfKttLearuWrP9MDH5MBPbIqV92AaeXatLxBI9gBaebbnrfifHhDYfgasaacH8akY=wiFfYdH8Gipec8Eeeu0xXdbba9frFj0=OqFfea0dXdd9vqai=hGuQ8kuc9pgc9s8qqaq=dirpe0xb9q8qiLsFr0=vr0=vr0dc8meaabaqaciaacaGaaeqabaqabeGadaaakeaacuWGrbqugaqeamaaBaaaleaacqWGPbqAcqWGUbGBcqWG0baDaeqaaaaa@324C@	Dinte MathType@MTEF@5@5@+=feaafiart1ev1aaatCvAUfKttLearuWrP9MDH5MBPbIqV92AaeXatLxBI9gBaebbnrfifHhDYfgasaacH8akY=wiFfYdH8Gipec8Eeeu0xXdbba9frFj0=OqFfea0dXdd9vqai=hGuQ8kuc9pgc9s8qqaq=dirpe0xb9q8qiLsFr0=vr0=vr0dc8meaabaqaciaacaGaaeqabaqabeGadaaakeaacqWGebardaqhaaWcbaGaemyAaKMaemOBa4MaemiDaqhabaGaemyzaugaaaaa@336E@	Q¯ MathType@MTEF@5@5@+=feaafiart1ev1aaatCvAUfKttLearuWrP9MDH5MBPbIqV92AaeXatLxBI9gBaebbnrfifHhDYfgasaacH8akY=wiFfYdH8Gipec8Eeeu0xXdbba9frFj0=OqFfea0dXdd9vqai=hGuQ8kuc9pgc9s8qqaq=dirpe0xb9q8qiLsFr0=vr0=vr0dc8meaabaqaciaacaGaaeqabaqabeGadaaakeaacuWGrbqugaqeaaaa@2DEF@	Q¯ MathType@MTEF@5@5@+=feaafiart1ev1aaatCvAUfKttLearuWrP9MDH5MBPbIqV92AaeXatLxBI9gBaebbnrfifHhDYfgasaacH8akY=wiFfYdH8Gipec8Eeeu0xXdbba9frFj0=OqFfea0dXdd9vqai=hGuQ8kuc9pgc9s8qqaq=dirpe0xb9q8qiLsFr0=vr0=vr0dc8meaabaqaciaacaGaaeqabaqabeGadaaakeaacuWGrbqugaqeaaaa@2DEF@	Q¯int MathType@MTEF@5@5@+=feaafiart1ev1aaatCvAUfKttLearuWrP9MDH5MBPbIqV92AaeXatLxBI9gBaebbnrfifHhDYfgasaacH8akY=wiFfYdH8Gipec8Eeeu0xXdbba9frFj0=OqFfea0dXdd9vqai=hGuQ8kuc9pgc9s8qqaq=dirpe0xb9q8qiLsFr0=vr0=vr0dc8meaabaqaciaacaGaaeqabaqabeGadaaakeaacuWGrbqugaqeamaaBaaaleaacqWGPbqAcqWGUbGBcqWG0baDaeqaaaaa@324C@	Dinte MathType@MTEF@5@5@+=feaafiart1ev1aaatCvAUfKttLearuWrP9MDH5MBPbIqV92AaeXatLxBI9gBaebbnrfifHhDYfgasaacH8akY=wiFfYdH8Gipec8Eeeu0xXdbba9frFj0=OqFfea0dXdd9vqai=hGuQ8kuc9pgc9s8qqaq=dirpe0xb9q8qiLsFr0=vr0=vr0dc8meaabaqaciaacaGaaeqabaqabeGadaaakeaacqWGebardaqhaaWcbaGaemyAaKMaemOBa4MaemiDaqhabaGaemyzaugaaaaa@336E@
2	89.4	90.7	87.5	46.7	90.1	90.1	87.2	47.3
3	93.0	92.7	89.0	50.5	93.0	92.7	89.9	56.4
4	97.9	96.7	93.5	60.7	97.9	97.2	94.6	68.2
6	98.0	97.0	93.7	63.5	98.0	97.7	95.4	69.1
20	99.1	97.6	93.9	67.3	98.7	98.2	94.8	69.1
20/Bastolla^*e*^	-	-	96.5	56.4	-	-	93.3	62.6
20/Lu^*e*^	-	-	93.6	63.6	-	-	93.5	63.5

^*a*^Results for the parameter sets produced with OS1. ^*b*^Results for the parameter sets produced with OS2. ^*c*^Results on the Optimization set. ^*d *^Results on the Test set. ^*e*^Q¯int
 MathType@MTEF@5@5@+=feaafiart1ev1aaatCvAUfKttLearuWrP9MDH5MBPbIqV92AaeXatLxBI9gBaebbnrfifHhDYfgasaacH8akY=wiFfYdH8Gipec8Eeeu0xXdbba9frFj0=OqFfea0dXdd9vqai=hGuQ8kuc9pgc9s8qqaq=dirpe0xb9q8qiLsFr0=vr0=vr0dc8meaabaqaciaacaGaaeqabaqabeGadaaakeaacuWGrbqugaqeamaaBaaaleaacqWGPbqAcqWGUbGBcqWG0baDaeqaaaaa@324C@ and *D*_*int *_include only the performance for interface recognition (the fold recognition statistics are left out). ^*e*^With the Bastolla or Lu energy functions.

The optimal energy parameters with the binary alphabet are consistent with the ones derived for fold recognition (Table 2). The parameters obtained using OS1 and OS2 are almost identical. Discrimination is fair, with a *D*_*int *_of 47% and a Q¯int
 MathType@MTEF@5@5@+=feaafiart1ev1aaatCvAUfKttLearuWrP9MDH5MBPbIqV92AaeXatLxBI9gBaebbnrfifHhDYfgasaacH8akY=wiFfYdH8Gipec8Eeeu0xXdbba9frFj0=OqFfea0dXdd9vqai=hGuQ8kuc9pgc9s8qqaq=dirpe0xb9q8qiLsFr0=vr0=vr0dc8meaabaqaciaacaGaaeqabaqabeGadaaakeaacuWGrbqugaqeamaaBaaaleaacqWGPbqAcqWGUbGBcqWG0baDaeqaaaaa@324C@ of 87% for both the OS1 and OS2 sets. The energy parameters with the ternary alphabet are consistent with the binary ones. Contacts between members of the two "hydrophobic" classes are stabilizing, for example. The discrimination power has increased: Q¯int
 MathType@MTEF@5@5@+=feaafiart1ev1aaatCvAUfKttLearuWrP9MDH5MBPbIqV92AaeXatLxBI9gBaebbnrfifHhDYfgasaacH8akY=wiFfYdH8Gipec8Eeeu0xXdbba9frFj0=OqFfea0dXdd9vqai=hGuQ8kuc9pgc9s8qqaq=dirpe0xb9q8qiLsFr0=vr0=vr0dc8meaabaqaciaacaGaaeqabaqabeGadaaakeaacuWGrbqugaqeamaaBaaaleaacqWGPbqAcqWGUbGBcqWG0baDaeqaaaaa@324C@ = 89% and *D*_*int *_= 53% (averaging over the OS1 and OS2 results).

With the four-class alphabet, Q¯int
 MathType@MTEF@5@5@+=feaafiart1ev1aaatCvAUfKttLearuWrP9MDH5MBPbIqV92AaeXatLxBI9gBaebbnrfifHhDYfgasaacH8akY=wiFfYdH8Gipec8Eeeu0xXdbba9frFj0=OqFfea0dXdd9vqai=hGuQ8kuc9pgc9s8qqaq=dirpe0xb9q8qiLsFr0=vr0=vr0dc8meaabaqaciaacaGaaeqabaqabeGadaaakeaacuWGrbqugaqeamaaBaaaleaacqWGPbqAcqWGUbGBcqWG0baDaeqaaaaa@324C@ and *D*_*int *_increase sharply, to 94% and 64%, respectively. Consistent with the monomeric results, contacts within and between the {LIVIMC} and {FYW} classes are the most stabilizing. Optimal parameters from OS1 and OS2 are similar. The rms deviation between the OS1 and OS2 energy matrices (with four amino acid classes) is 3.6 kcal/mol.

Performance improves only slightly with six classes: Q¯int
 MathType@MTEF@5@5@+=feaafiart1ev1aaatCvAUfKttLearuWrP9MDH5MBPbIqV92AaeXatLxBI9gBaebbnrfifHhDYfgasaacH8akY=wiFfYdH8Gipec8Eeeu0xXdbba9frFj0=OqFfea0dXdd9vqai=hGuQ8kuc9pgc9s8qqaq=dirpe0xb9q8qiLsFr0=vr0=vr0dc8meaabaqaciaacaGaaeqabaqabeGadaaakeaacuWGrbqugaqeamaaBaaaleaacqWGPbqAcqWGUbGBcqWG0baDaeqaaaaa@324C@ = 95% and *D*_*int *_= 66%. With the complete, 20-class alphabet, there is almost no further improvement: Q¯int
 MathType@MTEF@5@5@+=feaafiart1ev1aaatCvAUfKttLearuWrP9MDH5MBPbIqV92AaeXatLxBI9gBaebbnrfifHhDYfgasaacH8akY=wiFfYdH8Gipec8Eeeu0xXdbba9frFj0=OqFfea0dXdd9vqai=hGuQ8kuc9pgc9s8qqaq=dirpe0xb9q8qiLsFr0=vr0=vr0dc8meaabaqaciaacaGaaeqabaqabeGadaaakeaacuWGrbqugaqeamaaBaaaleaacqWGPbqAcqWGUbGBcqWG0baDaeqaaaaa@324C@ is 94% and *D*_*int *_is 68%. It appears that for the simple, residue–residue model used here, 4–6 amino acid classes are sufficient to encode the actual complexity of the model.

**Figure 6:**
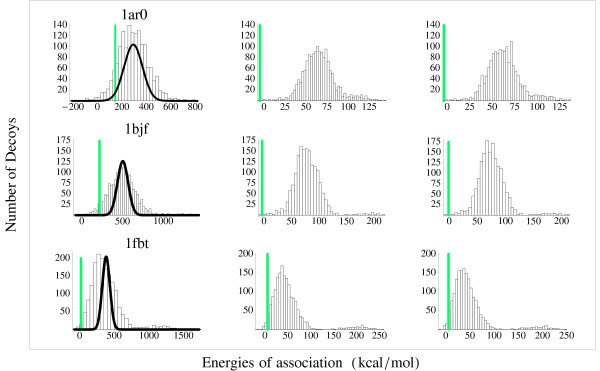
**Spectra of the distribution of the energy of association of different serie of decoys**. Native and decoy energy distributions for three complexes: 1AR0 (top), 1BJF (middle) and 1FBT (bottom), using the energy functions with two (left), six (middle), and 20 amino acid classes (right). The native energy is shown as a thin bar. Thick curves in the lefthand panels correspond to a random energy model for decoy energies; see main text. Our decoy energies are significantly more diverse than the random energy model.

To illustrate further the behavior of our energy functions, we consider the structures 1AR0, 1BJF, and 1FBT, which represent three typical situations. The energy spectra for the three decoy series are shown in Fig. 6, using the energy functions with two, six, and twenty amino acid classes.

• 1AR0: With 20 classes, discrimination is successful. The native complex is very stable compared to the decoys.

• 1BJF: The native complex is not recognized as the lowest-energy structure, but it has the third-best energy and is well-separated from the rest of the energy distribution (the decoys lying higher than it).

• 1FBT: This structure illustrates the worst case. The energy function is unable to recognize the native complex at any level of alphabet complexity. The native energy is quite high. The position of the native structure in the energy spectrum is only 0.5 standard deviations below the average decoy.

To illustrate the diversity of the decoy sets, we also calculated energy spectra with a "random energy" model. Decoys are not explicitly built; rather, we count the number of interface contacts in the native dimer A:B, say *N *contacts; we replace them by *N *contacts established between *N *random pairs, where the first amino acid is drawn from the surface of A and the second from the surface of B. Given the rather large numbers of interface constants, the corresponding energy spectra are essentially gaussian, with a standard deviation that is easy to compute from the composition in amino acid types (not shown). The distribution mean is taken to be the same as that of our decoy sets. The 2-class random energy spectra are shown in Fig. 6 (lefthand panels; thick lines). We see that our decoy sets lead to more diverse energies than the random energy model.

**Figure 7:**
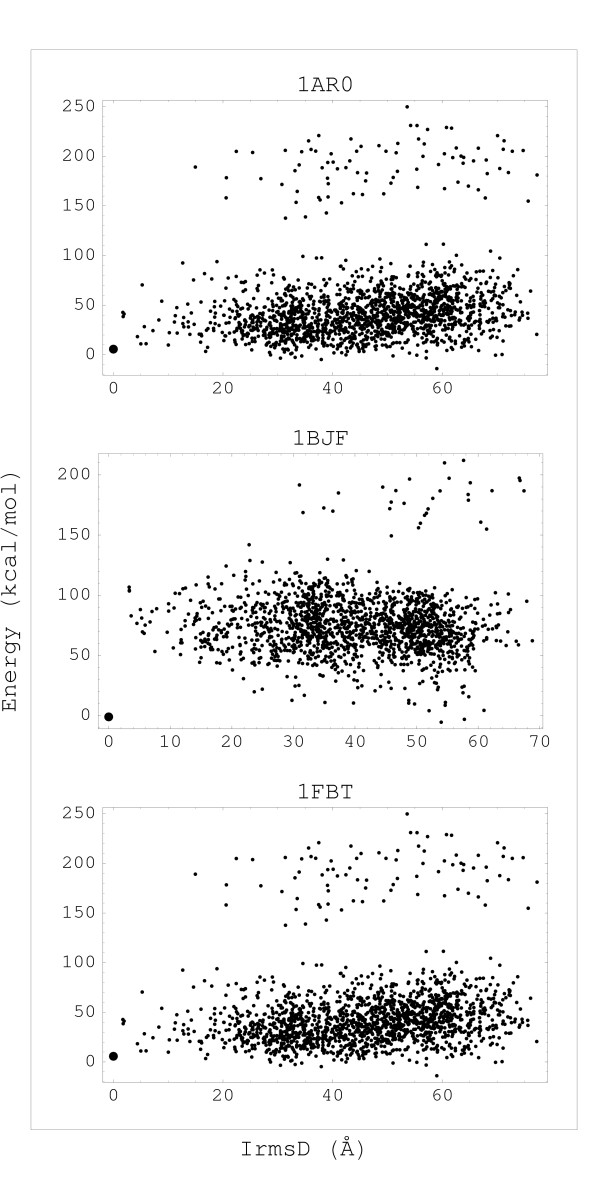
**Decoy energies versus deviation from native structure**. Decoy energies as a function of the structural deviation from native for three complexes: 1AR0 (top), 1BJF (middle) and 1FBT (bottom), using the energy function with 20 amino acid classes. The native energy is shown as a large dot (lower left). The structural deviation is measured by the rms difference RMSD in C_*α *_positions.

Finally, for 1AR0, 1BJF, and 1FBT, Fig. 7 illustrates our ability to discriminate decoys as a function of their deviations from the native structure. For the best case, 1ARO, there is a clear energy increase as one moves away from the native structure (large dot, lower left). For 1BJF, the native structure has a low energy, while the closest decoy structures are much higher. For 1FBT, the energy/structure correlation is fair, even though the native structure is not well-discriminated; the decoys that have a lower energy correspond to very different structures with a different, competing binding mode. Energy/structure correlations will be studied in more detail in future work. Notice that the decoy sets typically contain some, but not very many structures that are very close to native. This is not the goal of our docking approach, which focuses on the structural quality of the decoys (reasonable van der Waals and electrostatic interactions), the size of the interfaces (which should not be too small), and seeks to produce diverse interfaces. For the present class of energy functions, the number and nature of interface contacts are the most important decoy properties, and we believe these are adequately sampled with our procedure; this should lead to a successful parameterization. In contrast, we systematically include near-native structures in the blind testing of the energy functions further on.

#### Analysis of the energy parameters and amino acid classes

The simplified amino acid alphabets used here were derived from a clustering procedure based on the Blosum50 similarity matrix ("Blosum clustering"; see Methods, Eq. 4). An alternative classification can be obtained by performing a clustering based on the optimized energy matrix, constructed with all 20 amino acid types ("energy clustering"; Eq. 5). By comparing these two classifications, we can assess the robustness and consistency of the simplified alphabets. The two methods give somewhat different clusters, as shown in Fig. 1. At the lowest level, the energy function does not give a meaningful separation into two classes. It gives the same hydrophobic cluster as the Blosum50 matrix, with a good internal coherence (Pearson Correlation Coefficient of *R *= 0.58; Eq. 5). But the Blosum50 polar group is split in two by the energy clustering. At the six-class level, the two methods give the exact same amino acid groupings.

**Figure 8:**
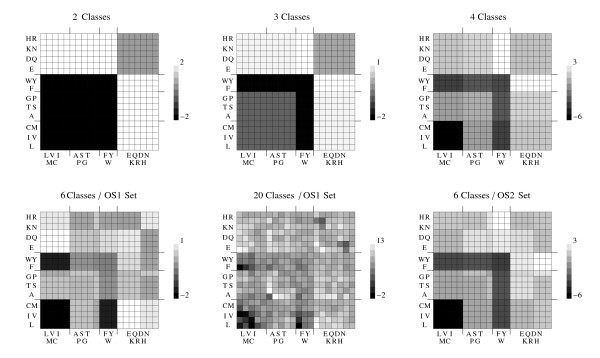
**Contour representations of the interaction parameters for selected energy functions**. Top row: energy functions with two, three and four amino acid classes, optimized for interface recognition using Optimization Set 2. Bottom row: six- and 20-class functions optimized over OS1 or OS2, as indicated. Contours levels in kcal/mol. Each energy matrix has a mean of zero. The amino acids belonging to each class are shown next to the corresponding rows and columns of each matrix. For the sake of clarity, the rows and columns of the 20-class matrix are not labelled individually but groupwise.

Fig. 8 illustrates the best energy function for each level of complexity, using a contour representation. The functions are seen, again, to form a coherent hierarchy. We note that with three, four, and six amino acid types, the best function was obtained independently of the simpler functions. Only the 20-class function was obtained using one of the simpler functions as a starting point (the 6-class/OS2 set, shown on the lower right). Thus, the consistency between the energy functions with the largest and the smaller alphabets is largely independent of the optimization procedure.

To further check the internal consistency of our hierarchy of energy functions, we take a "top-down" approach, using the complete, 20-class function as a starting point to infer simplified functions, which can be compared to the simplified functions described above. To infer a 6-class function, for example, we simply average the appropriate elements in the complete matrix. Thus, the lines and columns corresponding to K, R, and H in the complete 20 × 20 matrix form a submatrix, which is averaged to give the {KRH}{KRH} interaction in the inferred 6-class matrix. We also compute the standard deviation over the submatrix, which measures the internal consistency of the {KRH} subgroup within the complete matrix. Results for 4 and 6 classes are given in Table 5; results for 2 and 3 classes are qualitatively similar and are not shown. The simplified functions inferred in this way are in general agreement with the functions described above, obtained by direct optimization of Q¯
 MathType@MTEF@5@5@+=feaafiart1ev1aaatCvAUfKttLearuWrP9MDH5MBPbIqV92AaeXatLxBI9gBaebbnrfifHhDYfgasaacH8akY=wiFfYdH8Gipec8Eeeu0xXdbba9frFj0=OqFfea0dXdd9vqai=hGuQ8kuc9pgc9s8qqaq=dirpe0xb9q8qiLsFr0=vr0=vr0dc8meaabaqaciaacaGaaeqabaqabeGadaaakeaacuWGrbqugaqeaaaa@2DEF@.

**Table 5:** Four- and six-class parameters averaged over the complete energy matrix (standard deviation in parentheses) In Kca/mol.

	H_*α*_	H_*β'*_	H_*γ*_	P_*α*_	P_*β*_	G	
	-2.03(0.07)	0.13(0.06)	-1.70(0.05)	0.79(0.04)	0.54(0.04)	-0.13(0.03)	H_*α*_
		0.01(0.05)	-0.43(0.05)	0.16(0.05)	-0.22(0.05)	0.11(0.04)	H_*β'*_
H_*α*_	-2.03(0.07)		-0.65(0.02)	0.37(0.05)	-0.22(0.06)	-0.56(0.06)	H_*γ*_
H_*β*_	0.08(0.11)	0.05(0.07)		0.55(0.05)	-0.27(0.07)	0.03(0.07)	P_*α*_
H_*γ*_	-1.70(0.05)	-0.45(0.07)	-0.65(0.02)		0.56(0.08)	0.24(0.13)	P_*β*_
P	0.71(0.10)	0.02(0.19)	0.12(0.30)	0.15(0.42)		0.1(0.)	G
	H_*α*_	H_*β*_	H_*γ*_	P			

### Evaluating the energy functions in blind tests

#### A test set of heterodimers

Our Optimization and Test sets are mostly made up of homodimers, due to the relative scarceness of heterodimers in the PDB (excluding trimers and higher order multimers). To assess the effect of this, we set aside for a blind test 23 heterodimers that are not homologous to any structures used in the parameterization. Table 6 gives the performance for these sets of our best energy functions, as well as the Bastolla function. Discrimination is about 54% (13/24), compared to about 68% for the homodimers (Table 4). (Cross-validated) results are also listed for the heterodimers in the two optimization sets and are quite similar (8/17 and 9/17 with the 20- and 6-class functions, respectively). The weaker discrimination *D*_3 _gives a success rate of about 71% (17/24 series for which the native structure is among the three best energies; see Methods). Clearly, it is easier to discriminate the homodimers, which usually have larger interfaces and stronger binding constants. A similar behavior and discrimination quality are seen below for the other blind tests.

**Table 6:** Energy rank for heterodimeric structures. Energy rank of the native structure, compared to its decoys, using various energy functions. ^*a*^23 hetermodimers with their decoys, not used in the parameterization. The 20- and 6-class energy functions used are the ones optimized on OS2. ^*b*^Heterodimers in the TS1 and TS2 data sets. For the structures in TS1 (bottom 11), the OS1 energy functions are used; for those in TS2 (top 6), the OS2 functions are used (i.e., we show cross-validated results). ^*c*^Fraction of successful series. *D *and *D*_3 _correspond to strong discrimination (native structure ranked first) and weak discrimination (native ranked among top three; see Methods).

**Blind heterodimer set^*a*^**				**TS1, TS2 sets^*b*^**			
PDB ID	20Cl	Bastolla	6Cl	PDB ID	20Cl	Bastolla	6Cl
1ai7	1	1	1	1abr	4	2	3
1as4	1	1	1	1aui	1	1	1
1bpl	1	1	1	1ay7	3	3	2
1clv	2	1	2	1blx	3	6	3
1dj7	4	4	4	1c1y	3	4	3
1dow	1	1	1	1dkf	2	47	1
1eud	8	9	7	1ugh	1	3	1
1f0c	1	1	1	1efv	1	1	1
1ksg	5	3	5	1ezq	1	1	1
1ku6	3	2	4	1f3v	67	145	36
1lot	1	1	1	1fle	19	4	9
1lpb	20	8	16	1hdm	1	1	1
1mhm	1	1	1	1itb	1	1	3
1mtp	1	1	1	1phn	1	1	1
1mzw	3	3	4	1qav	1	2	1
1npe	4	4	2	1smp	14	69	2
1nw9	1	10	1	1stf	2	5	1
				
1n52	10	1	9	*D*^*c*^	8/17	6/17	9/17
1o5m	3	1	2	*D*_3_	13/17	9/17	15/17
				
1qge	1	1	1				
1rke	1	1	1				
1rtj	1	2	1				
1ubt	1	2	1				
4sgb	5	6	5				
				
*D*^*c*^	13/24	13/24	13/24				
*D*_3_	17/24	18/24	16/24				

#### Decoys constructed by other methods: Sternberg and Vakser decoys

Limited series of protein–protein structures have been made available by Sternberg (ten series) and Vakser (five series) and coworkers, along with 99 high-quality decoys each. They are listed in Supplementary Material: see additional file 3, Supp3.ps. These series have been used in the past to assess energy functions, including one function of the same form as ours [19,32] and some more complicated ones [19,21]. We use them for a blind test of our energy functions. With the Sternberg structures, our best energy function ranked the native complex among the top ten structures in just three cases out of ten (Table 7). Our best function is the 20-class function optimized using OS2 (see Table 4); the 20-class function optimized using OS1 gave slightly poorer results. Very poor scoring occurred for three complexes (99–100th position for the native structure). The 1AVZ complex, for example, has the lowest interface similarity between the top decoys and the native structure (as measured by the fraction of common contacts). Its native interface is unusually small, with an area of just 1076 Å^2 ^and only 28 interresidue contacts across the interface. The other failures were for 1BRC, 1BGS, 1DFJ, 1WQ1, 2PCC and 2SIC. For IBRC, two near-native structures were ranked among the top five. These decoys have rms deviations of just 1.6 and 1.9 Å from the native complex, so that 1BRC can actually be considered a near-success. For 1BGS, the parameters optimized using OS1 correctly ranked the 1BGS experimental structure first. For 1UGS, 2PCC and 2SIC, a near-native decoy is highly-ranked (3rd, 7th and 3rd, with rmsd's of 5.3, 4.6 and 3.2, Å respectively). For WQ1, the native structure is ranked last; however, decoys 4 and 5 are ranked 6th and 10th. 1DFJ gives the worst results. The native structure is ranked last by our energy functions. No decoy ressembling the native structure is ranked among the top ten structures. 1DFJ has a native interface that is poor in hydrophobic residues (only 27% of the contacts). In contrast, some of its decoys have interfaces rich in hydrophobic residues.

**Table 7:** Blind tests of dimer interface recognition: the rank of the native structure. ^*a*^The top (1AVZ-2SIC) and middle groups are the Sternberg and Vakser test sets, respectively (see Methods). The lower group (T04–T19) are the 2005 CAPRI target structures. ^*b*^Columns 2–5 correspond to the energy functions optimized with OS1 and OS2, using 20, 4, or 6 amino acid classes, as indicated.^*c*^Energy functions from Refs. [19], [20]. Numbers in bold show cases where the native structure is ranked among the top ten structures.

PDB ID^*a*^	20Cl OS1^*b*^	20Cl OS2	6Cl OS2	4Cl OS2	Lu^*c*^	Bastolla^*c*^
1AVZ	73	35	67	100	54	40
1BGS	**1**	65	62	39	16	**1**
1BRC	76	37	41	31	**5**	70
1CGI	56	**1**	**1**	**1**	**1**	18
1DFJ	100	100	100	100	78	100
1FSS	24	**1**	**1**	**1**	**1**	**1**
1UGH	11	**10**	20	**10**	**10**	71
1WQ1	99	100	100	100	97	98
2PCC	20	72	82	79	25	23
2SIC	17	48	56	17	14	96

5CHA-2OVO	**6**	**7**	**3**	**2**	**2**	**3**
2PTN-4PTI	**5**	**9**	**9**	33	**4**	**10**
1SUP-2CI2	**4**	**4**	**4**	**4**	**5**	**9**
1A2P-1A19	31	58	52	27	20	17
1CHG-1HPT	**2**	**7**	**4**	**6**	**2**	**4**

T04	**9**	**8**	**10**	11	11	**1**
T05	64	61	62	62	60	60
T06	**2**	**3**	**3**	**1**	**4**	**3**
T07	58	63	62	56	58	64
T08	84	52	68	96	136	145
T09	165	162	164	165	159	164
T11	**1**	**1**	**2**	**1**	**1**	**1**
T12	**9**	**5**	**6**	**2**	**1**	**5**
T13	194	176	179	175	174	175
T14	68	121	91	125	18	43
T15	12	**3**	**4**	**7**	**1**	**1**
T18	25	**8**	**7**	**1**	**2**	**4**
T19	**3**	38	22	**1**	37	**6**

Our energy function was more successful with the Vakser set, with one failure and four successes (native structure among the top ten). One of the successful Vakser targets (1CHG-1HPT) is also part of the Sternberg set (labelled 1CGI in Table 7; also successful). However, part of the structure is missing in the Vakser complex (14 amino acids) and the decoy sets are different; therefore, we consider the Vakser target to be a separate test. Overall, we find that our energy function behaves most successfully when the native interface has a fairly typical hydrophobic/hydrophilic profile. This presumably reflects the predominance of homodimers in the training sets (see above).

We also did calculations with two energy functions developed by Bastolla et al [20] and Lu et al [19]. With our own Test Sets and the Bastolla function, we obtained *D*_*int *_= 56.4% and Q¯int
 MathType@MTEF@5@5@+=feaafiart1ev1aaatCvAUfKttLearuWrP9MDH5MBPbIqV92AaeXatLxBI9gBaebbnrfifHhDYfgasaacH8akY=wiFfYdH8Gipec8Eeeu0xXdbba9frFj0=OqFfea0dXdd9vqai=hGuQ8kuc9pgc9s8qqaq=dirpe0xb9q8qiLsFr0=vr0=vr0dc8meaabaqaciaacaGaaeqabaqabeGadaaakeaacuWGrbqugaqeamaaBaaaleaacqWGPbqAcqWGUbGBcqWG0baDaeqaaaaa@324C@ = 96.5% for Test Set 1, and *D*_*int *_= 62.6% and Q¯int
 MathType@MTEF@5@5@+=feaafiart1ev1aaatCvAUfKttLearuWrP9MDH5MBPbIqV92AaeXatLxBI9gBaebbnrfifHhDYfgasaacH8akY=wiFfYdH8Gipec8Eeeu0xXdbba9frFj0=OqFfea0dXdd9vqai=hGuQ8kuc9pgc9s8qqaq=dirpe0xb9q8qiLsFr0=vr0=vr0dc8meaabaqaciaacaGaaeqabaqabeGadaaakeaacuWGrbqugaqeamaaBaaaleaacqWGPbqAcqWGUbGBcqWG0baDaeqaaaaa@324C@ = 93.3% for Test Set 2. With the Lu et al function, we obtained *D*_*int *_= 63.6% and Q¯int
 MathType@MTEF@5@5@+=feaafiart1ev1aaatCvAUfKttLearuWrP9MDH5MBPbIqV92AaeXatLxBI9gBaebbnrfifHhDYfgasaacH8akY=wiFfYdH8Gipec8Eeeu0xXdbba9frFj0=OqFfea0dXdd9vqai=hGuQ8kuc9pgc9s8qqaq=dirpe0xb9q8qiLsFr0=vr0=vr0dc8meaabaqaciaacaGaaeqabaqabeGadaaakeaacuWGrbqugaqeamaaBaaaleaacqWGPbqAcqWGUbGBcqWG0baDaeqaaaaa@324C@ = 93,6% for Test Set 1, and *D*_*int *_= 63.5% and Q¯int
 MathType@MTEF@5@5@+=feaafiart1ev1aaatCvAUfKttLearuWrP9MDH5MBPbIqV92AaeXatLxBI9gBaebbnrfifHhDYfgasaacH8akY=wiFfYdH8Gipec8Eeeu0xXdbba9frFj0=OqFfea0dXdd9vqai=hGuQ8kuc9pgc9s8qqaq=dirpe0xb9q8qiLsFr0=vr0=vr0dc8meaabaqaciaacaGaaeqabaqabeGadaaakeaacuWGrbqugaqeamaaBaaaleaacqWGPbqAcqWGUbGBcqWG0baDaeqaaaaa@324C@ = 93.5% for Test Set 2. These results (Table 4) are somewhat poorer than the ones obtained with our own functions, except for the Bastolla Q¯int
 MathType@MTEF@5@5@+=feaafiart1ev1aaatCvAUfKttLearuWrP9MDH5MBPbIqV92AaeXatLxBI9gBaebbnrfifHhDYfgasaacH8akY=wiFfYdH8Gipec8Eeeu0xXdbba9frFj0=OqFfea0dXdd9vqai=hGuQ8kuc9pgc9s8qqaq=dirpe0xb9q8qiLsFr0=vr0=vr0dc8meaabaqaciaacaGaaeqabaqabeGadaaakeaacuWGrbqugaqeamaaBaaaleaacqWGPbqAcqWGUbGBcqWG0baDaeqaaaaa@324C@ with Test Set 1. The correlation coefficient between our OS2 20-class function and the Bastolla function is 0.70. For the 15 Sternberg and Vakser blind tests, the Bastolla and Lu functions gave six and eight clear successes, compared to seven with our best function (eight, including 1BRC; Table 7). Performance for near-native decoys was qualitatively similar for all three functions.

With the Sternberg decoys, there are small internal deformations of the two partners, relative to the experimental dimer structure. Here, we take these into account, scoring each structure by its binding free energy. In contrast, Lu et al [19] employed the interface free energy, which ignores internal deformation. This leads to a better ranking of the native structure, but is physically questionable, since dimerization is governed by the total binding free energy, and not the interface free energy alone.

Finally, we note that our best 4- and 6-class functions, remarkably, gave results of practically the same quality as the 20-class functions (Table 7).

#### Structures submitted to the CAPRI experiment

The Capri structure sets are expected to be the most difficult test. Indeed, the structures submitted are not decoys in the usual sense, but attempts by the participating groups to predict the target structure. All the target structures were determined by Xray crystallography, with an experimental resolution of 3.0 Å or better. We considered 13 of the 19 CAPRI targets. The others were discarded for technical reasons (eg, gaps or insertions in the decoy structures). In Tables 7, 8, we report results with two 20-class energy functions, optimized using OS1 and OS2, respectively. The OS2 function gave distinctly better results for our own structure sets (Table 4) and for the Sternberg blind tests, above. Therefore, we take this as our best energy function.

In four cases, T06, T11, T12 and T15, our best energy function correctly identified the native complex within the five lowest-energy structures. In two cases, T04 and T18, the experimental complex was ranked within the top ten. T04, T05, and T06 are complexes between an alpha-amylase and three different camelide antibodies. T05 has slightly fewer polar–polar contacts than T04 and T06. T11 and T12 correspond to the same cohesin-dockerin complex. For T11, CAPRI participants received the unbound cohesin crystal structure and the NMR structure of a homologous dockerin domain, sharing 50% sequence identity with T11. The decoys thus involve a homology-modelling of the T11 dockerin moiety. For T12, they received the same, unbound cohesin structure, while the dockerin moiety was from the crystal structure of the complex. Although the two complexes correspond to the same native interface, their sets of decoys, submitted by the CAPRI participants, are completely different. Therefore, we count them as separate targets. The native interfaces for T06 and T11/T12 feature similar kinds of interactions, with 45% of hydrophobic-hydrophobic contacts (the {LVIMCGPTASFYW} class), 43% of polar–polar contacts (the {EDNQKRH} class), and 12% of polar–hydrophobic contacts (averaged over the 3 structures). The percentage of interface contacts of each type are given in Table 8, along with the total number of contacts, relative to the native structure. T11 and T12 had 190 and 214 decoys, respectively. Our successes for T15 (Colicin D catalytic domain-Colicin D immunity protein complex) and T18 (a xylanase–inhibitor complex) are notable, as their interface hydrophobicities are distinctly untypical, with a predominance of hydrophobic–polar interactions and a large number of polar–polar interactions.

**Table 8:** Native complex discrimination and residue contacts at the interfaces of submitted and target CAPRI structures

		Interface contacts^*b*^			
Target number	Native rank^*a*^	HH	HP	PP	Top decoy's interface contact number *F*^*c*^
T04	**9 8**	53 (49.8)	32 (39.7)	15 (10.4)	1.1
T05	64 61	42 (56.8)	51 (34.4)	7 (8.7)	1.4
T06	**2 3**	46 (43.1)	33 (37.1)	21 (19.9)	1.0
T07	58 62	23 (25.7)	43 (44.9)	35 (29.3)	1.3
T08	84 61	7 (40.2)	77 (42.6)	16 (17.1)	4.3
T09	165 162	27 (39.2)	53 (42.1)	20 (18.6)	2.2
T11	**1 1**	48 (42.5)	41 (42.8)	11 (14.6)	1.0
T12	**9 5**	48 (53.5)	41 (36.9)	11 (9.5)	1.1
T13	194 176	65 (64.2)	33 (30.5)	2 (5.3)	1.0
T14	68 121	26 (31.4)	51 (44.2)	23 (24.4)	0.6
T15	12 **3**	18 (17.3)	44 (48.6)	38 (14.5)	0.8
T18	25 **8**	36 (49.3)	49 (43.1)	14 (7.6)	1.0
T19	**3 **38	36 (45.1)	44 (40.0)	20 (14.9)	0.6

^*a*^Values for the 20Cl/OS1, OS2 functions, already given in Table 7. ^*b*^The percentage of interface contacts of each type, averaged over the 99 decoy structures: hydrophobic-hydrophobic (HH), hydrophobic-polar (HP), polar–polar (PP); values for the native structure in parentheses. ^*c*^The relative contact number *F *= *N*_*dec*_/*N*_*nat *_(Eq. 2) of the decoy ranked first by the 20-class OS2 energy function.

Among the failures, T05 is the worst case. Note that none of the models submitted by the CAPRI participants for T05 (or T04) were acceptable. Thus, the 66 T05 "decoys" included only a few structures that were at all similar to the native (eg, only nine have at least one of the native interface contacts). The best "decoy" submitted for T05 was ranked 29th by our energy function. The T07, T08 and T09 interfaces are rather poor in hydrophobic contacts (from 26 to 40% of the interface contacts). Their decoy sets all include interfaces with more typical hydrophobic contents. This may explain the poor ranking of the native structure for these three targets (62th out of 71, 73rd out of 180, 162nd out of 165 for T07, T08, T09, respectively).

The other three failed trials, T13, T14 and T19, correspond to large decoy sets (211, 251 and 237 submitted structures), all of a very good quality (as judged by the Charmm19 energy function, for example). The T13 set includes 53 submitted complexes that present at least one native contact. Among the decoys closest to the native structure, the best one (contact similarity *q *of 0.87; *i.e*., 87% of its contacts are native-like) is still poorly ranked by our energy function (44th position). The T14 decoy set is of an even better quality, with half of its 251 structures featuring some native contacts. In this case, the decoy that is closest to the native structure is ranked 18th by our best function. Our result for the T19 complex (an ovine prion–Fab complex) is somewhat better. The structure that is closest to the experimental structure (with a contact similarity *q *of 0.53) is ranked 2nd by our energy function. Overall, the Bastolla and Lu energy functions performed in a similar fashion (Table 7). They both failed to correctly rank T05, T07, T08, T09, T13 and T14.

Finally, to illustrate further the occurrence of false positive and false negative predictions, we considered the nine CAPRI series that contain at least one near-native decoy (T06, 7, 8, 12, 13, 14, 15, 18, 19). For six of these, we ranked at least one near-native decoy among the top ten structures. In all, with the 20-class function, 27% of the native or near-native structures were recognized (highly ranked); 30% were recognized with the 6-class function.

## Discussion

### The quality of interface recognition

An important goal of modern genomics is to identify interacting proteins and characterize the structure and function of the corresponding complexes. A promising strategy is to assemble putative complexes from individual protein structures and characterize them by computing a binding free energy. Here, we have developed simple, empirical energy functions for interface recognition. We used a coarse-grained description, at the residue level, along with the simplest possible, stepwise, distance-dependence. More compex models are known to give superior results [10,18,19,21,26,33]. Here, however, we pursued two more limited goals. First, we examined whether a large computational effort would lead to better parameterization and performance of this simplest class of models. Second, we examined what size amino acid library is needed for optimal performance.

Thanks to increased computing power, we employed many more structures and many more decoys than previous workers. The decoys were both diverse and structurally-realistic (for example, in terms of interface size), thanks to a realistic, flexible docking method of construction. In particular, van der Waals and electrostatic contacts between partners are reasonable. The decoy set was characterized in some detail; more details will be given elsewhere. Our search of parameter space was also considerably more thorough than some past work. This is important for the 6-, and especially the 20-class amino acid alphabets, which have 21 and 210 adjustable parameters, respectively. For these, we considered a total of over 140,000 starting guesses for the parameters. We also included fold recognition in the optimization criterion (using additional sets of target structures and decoys), which should help to restrain the parameters to physically meaningful values. Finally, by constructing a hierarchy of energy functions, with amino acid alphabets of increasing complexity, we could focus the parameter search effectively for the larger alphabets. For example, our best 20-class function was obtained using a 6-class function as a starting point. The performance of our method is measured by its ability to identify the experimental, native interface from among sets of decoys.

Despite the improvements made here, the performance of our functions is very similar to those (of the same complexity) developed by Bastolla, Lu, and coworkers. For the CAPRI tests, we had six successes out of 13 targets with our 6- and 20-class functions, while our 4-class function actually gave a seventh near-success (the native T04 structure, ranked 11th). The Bastolla function gave seven successes and the Lu function, six. The successful cases are almost the same with all the functions, and behavior for near-native decoys is similar. This is a bit disappointing, given the extensive computational effort in the present work. However, this effort was needed to establish firmly that the performance limit of this class of models has been reached.

### A coherent hierarchy of amino acid alphabets and models

Our second goal was to examine a hierarchy of amino acid alphabets and models. This analysis has produced new insights into both the similarities and differences among amino acids, and the diversity required for protein–protein recognition. The amino acid groupings were initially obtained by clustering the Blosum50 similarity matrix [30], which is itself based on the mutational frequencies seen in multiple sequence alignments [34]. The mutational frequencies are mainly determined by factors other than interface recognition. Presumably, the dominant mutational constraint is the fold and stability of the individual protein [35]. Protein–protein recognition is one of several secondary constraints, along with folding kinetics and robustness towards mutations [36]. Nevertheless, the Blosum clustering leads to good quality interface recognition. At the 4-class level, for example, two thirds of the native structures in our data set are ranked first, among over 1300 high-quality decoys. This is consistent with the idea that similar physical-chemical effects operate in protein folding and protein–protein association.

The best energy functions at each level were obtained from random initial guesses, except for the 20-class function, which was obtained from a 6-class function. Nevertheless, the energy functions form a consistent hierarchy, with qualitatively similar parameters at all levels. This is especially striking in the contour representation of Fig. 8. From the complete energy matrix, we can also infer amino acid groupings (left of Fig. 1). The 20-class matrix gives back the Blosum 6-class grouping, as expected (because of its construction from a 6-class function). At the 4-class level, there is an interesting difference between the two groupings. Blosum splits the aromatics {FWY} into their own group, whereas the energy matrix classification splits the polar amino acids into two groups, based partly on electrostatic charge: {DENQ} and {HKR}. At the 6-class level, polar–polar interactions are repulsive, except for the {DENQ}{HKR} interaction, which is reasonable. On the other hand, F, W, and Y have much higher interface propensities than the other hydrophobics except Met, so that an aromatic group is also reasonable. The 6-class function captures both of these effects. The internal coherence of the 4- and 6-class energy groupings is very high (see correlation coefficients of each group, left of Fig. 1). In future work, it would be interesting to also consider 5-class functions.

## Conclusion

The good performance of the 4- and 6-class functions, compared to the 20-class function, may be partly due to the limited structural data available today for parameterization. If the number of known dimeric structures (particularly heterodimers) were to increase substantially, there may be enough information to parameterize a superior 20-class function. Nevertheless, the good performance with four and six classes suggests that a moderate amino acid diversity is sufficient to establish specific protein–protein recognition. One might speculate that this facilitated the emergence of protein–protein interaction networks in the course of evolution. Indeed, it seems much easier to perform a "coarse-grained" optimization of an interface, rather than a "detailed" optimization; *i.e*., it seems easier to optimize the distribution of interface residues among a limited set of four or six groups, rather than optimizing the exact amino acid distribution at the interface.

The present models can serve as the basis for future, more complex models that use a reduced amino acid library but include additional physical effects [22]. More complex models are needed to detect transient complexes, which are likely to be missed by the present, coarse-grained model. An improved model could account for the distance dependency of electrostatic interactions, either between polar residues or between polar residues and solvent. Backbone contacts between protomers are another possibility. With twenty amino acid types, such models lead to a very large number of adjustable parameters. A high-quality, 6-class grouping could provide a better starting point for a model that balances efficiency and realism. The decoys sets created here are available on request and will also be of use, especially as flexible docking strategies become more common.

The models developed here have already proven useful for the inverse protein folding problem (unpublished data). Their good performance for the difficult CAPRI tests suggests that they will also be useful for exploring protein–protein interactions at the proteome scale, particularly for strongly interacting protein–protein complexes such as those used in the model parameterization. Improved, more sophisticated models will have an increased ability to detect the weaker, transient complexes that are also common in the proteome.

## Methods

### Monomeric structure selection

We used 810 X-ray structures from the PDBselect collection [37]. Sequence identity between all pairs was less than 25%. The structures were monomeric and did not contain any cofactors or metal ions. Crystallographic resolution was better than 3.3 Å (average of 2.0 Å). They were divided into two sets, having similar average chain lengths (around 190 amino acids). 615 proteins were used as an Optimization set, for the parameterization of the energy functions below. The other 200 were used as a Test set, for evaluating the parameters' performance.

### Monomeric decoys

For each native structure, we performed a gapless threading of the sequence onto segments of matching size from all the structures in our 810 protein set. On average, we obtained 349 decoys per native structure. For each amino acid position in a decoy, the most probable rotamer for the given amino acid type was arbitrarily taken from the Dunbrack backbone-independant rotamer library [38] and constructed. Thus, steric constraints could be violated in the decoy structures, because the rotamers were not optimized with respect to the particular backbone and sidechain environment. With the energy functions used below, this is not a difficulty. Furthermore, this simple method for constructing monomeric decoys was deemed sufficient, because our main focus is not fold recognition, but the identification of protein–protein complexes.

### Dimeric structure selection

We used 219 dimer structures from the DIMER-1 set of Lu et al [19], which contains 340 structures. We retained only dimers, as opposed to higher-order complexes. This distinction is not always straightforward, and one higher-order complex was identified in the data set at a late stage. It is not expected to affect the results, judging by the similar parameters obtained with OS1 and OS2, or when an optimized parameter set is refined further with the higher-order structure left out. All the structures have a crystallographic resolution better than 2.5 Å, 30 interface contacts or more (using a 4.5 Å distance cutoff), and a pairwise sequence identity with any other member of the set below 35%. All correspond to biological dimers, as opposed to crystal contacts. This was established by running an automated test with the PQS server [39] and by checking the literature.

Our final set included 195 homodimers and just 17 heterodimers. A second set of 23 heterodimers was set aside to perform blind tests after parameterization was finished. The predominance of homodimers was largely imposed by the structures available in the PDB [40]. A typical homodimer is expected to have a larger, somewhat more hydrophobic interface, and a stronger binding constant, compared to a typical heterodimer. This is both a limitation and an advantage for our purposes. We expect that there will be a stronger dimerization signal in the homodimer interfaces and, furthermore, the signal is repeated twice, since the complexes are almost always symmetrical. This should facilitate parameterization, without damaging the heterodimer performance. Indeed, heterodimer association is driven by largely the same physical effects as homodimerization, so that the easier homodimer parameterization should carry over and be applicable to heterodimers. This is confirmed by our blind tests on the separate set of 23 heterodimers, which are not used in any step of the parameterization, and are not homologous to any of the proteins used.

### Dimeric decoys

Decoy sets were built by a docking procedure, starting from each native, dimeric structure. First, the two, native partners were shifted apart through a random translation and rotation of one of the partners. Second, rigid body minimization was done, using the Charmm19 molecular mechanics energy function [41]. No solvent model was included; ie, electrostatic interactions were computed with a dielectric constant of one. A harmonic pulling restraint was applied between the two centers of masses. The target distance was the native separation distance; the force constant was 60 kcal/mol/Å^2^. After 100 steps of rigid body minimization, the number of interprotein contacts was evaluated as the number of pairs of residues with at least one interatomic distance below 4.5 Å. We then compared the number of interprotein contacts in the decoy and the native complex. We define the "relative contact number" *F *of a given structure by:

F=NdecNnat,
 MathType@MTEF@5@5@+=feaafiart1ev1aaatCvAUfKttLearuWrP9MDH5MBPbIqV92AaeXatLxBI9gBaebbnrfifHhDYfgasaacH8akY=wiFfYdH8Gipec8Eeeu0xXdbba9frFj0=OqFfea0dXdd9vqai=hGuQ8kuc9pgc9s8qqaq=dirpe0xb9q8qiLsFr0=vr0=vr0dc8meaabaqaciaacaGaaeqabaqabeGadaaakeaacqWGgbGrcqGH9aqpdaWcaaqaaiabd6eaonaaBaaaleaacqWGKbazcqWGLbqzcqWGJbWyaeqaaaGcbaGaemOta40aaSbaaSqaaiabd6gaUjabdggaHjabdsha0bqabaaaaOGaeiilaWcaaa@3A81@

where *N*_*dec*, _*N*_*nat *_are the number of residue–residue contacts in the decoy and native structure, respectively. If *F *was at least 80%, the decoy coordinates were output at this stage. If not (the vast majority of cases), minimization was continued, without the pulling restraint, but allowing now intramolecular deformation of one of the partners. Also, an electrostatic contrast was introduced by enhancing the atomic charges on the two partners (with respect to the Charmm19 values), to increase the interprotein attraction. The charges of one partner were all increased by 0.25*e*, while the charges of the other partner were decreased by 0.25*e*. Intraprotein sidechain–sidechain electrostatic interactions were omitted and intraprotein sidechain-backbone electrostatic interactions were reduced by 1/2.

After 50 steps of Powell minimization [42], we compared the decoy structure to the native one. For the "deformable" partner, we performed a best fit of the monomer on its native structure and evaluated the "intramolecular" rms deviation between the two, considering its sidechains only. If the sidechain deviation was above 4.5 Å, the decoy was discarded. Decoys with *F *< 0.45 were also discarded. Decoys with a van der Waals energy greater than 4000 kcal/mol were also discarded. If the decoy was too similar to the native complex it was discarded. The criterion for similarity was the rms deviation for the "mobile" partner in the decoy and the native complex: if the rms deviation was less than 3 Å, the decoy was discarded. The deviation was computed without superimposing the two structures, so that it measures the overall motion of the mobile partner away from its starting, native position. Finally, to eliminate redundant decoy structures, we computed rms deviations between all pairs of decoy structures, rejecting decoys that were within 3.5 Å of another decoy. In all, we obtained a total of about 290000 decoys, for 219 native complexes, giving an average of 1326 decoys per native complex, with a minimum of 277 and a maximum of 1843 decoys. Typically, several tries were needed per decoy, so that the first step of the protocol (random displacement of one partner) was done over one million times.

To assess the quality of decoy structures, we used a detailed, atomic energy function, corresponding to the Charmm19 molecular mechanics force field [41]. The solvent environment was modelled with the ACE variant [43] of the Generalized Born model, parameterized as in [44]. These calculations were done with the XPLOR program [45].

### Empirical, residue-based energy function

Consider a protein with the amino acid sequence *S *and a given three-dimensional conformation. We use a residue-based energy function, of the form:

E(C)=∑i,jCijU(Si,Sj)
 MathType@MTEF@5@5@+=feaafiart1ev1aaatCvAUfKttLearuWrP9MDH5MBPbIqV92AaeXatLxBI9gBaebbnrfifHhDYfgasaacH8akY=wiFfYdH8Gipec8Eeeu0xXdbba9frFj0=OqFfea0dXdd9vqai=hGuQ8kuc9pgc9s8qqaq=dirpe0xb9q8qiLsFr0=vr0=vr0dc8meaabaqaciaacaGaaeqabaqabeGadaaakeaacqWGfbqrcqGGOaakcqWGdbWqcqGGPaqkcqGH9aqpdaaeqbqaaiabdoeadnaaBaaaleaacqWGPbqAcqWGQbGAaeqaaaqaaiabdMgaPjabcYcaSiabdQgaQbqab0GaeyyeIuoakiabdwfavjabcIcaOiabdofatnaaBaaaleaacqWGPbqAaeqaaOGaeiilaWIaem4uam1aaSbaaSqaaiabdQgaQbqabaGccqGGPaqkaaa@447A@

where the sum is over all pairs of amino acids *i*, *j *in the protein. *U*(*S*_*i*_, *S*_*j*_) is an interaction energy, which depends on the amino acid types *S*_*i*_, *S*_*j *_at positions *i*, *j*. *C*_*ij *_is one if the pair *i*, *j *is close together, and zero otherwise. Specifically, *i*, *j *are assumed to interact if they have at least one pair of nonhydrogen atoms within a distance of 4.5 Å, and if they are not first or second neighbors along the polypeptide chain; i.e., |*i *– *j*| ≥ 3. We refer to *U *as an energy matrix, and to *C *as a "contact map".

With a complete amino acid alphabet of twenty types, the 210 elements in the energy matrix *U *are all different. With a reduced alphabet, amino acid types belonging to the same group share the same coefficients in the energy matrix *U*. For example, with a binary, hydrophobic/hydrophilic alphabet, the 210 elements in *U *fall into just three categories, and have just three possible values, corresponding to hydrophobic–hydrophobic, hydrophobic–hydrophilic, or hydrophilic–hydrophilic pairs. In what follows, we consider alphabets with two, three, four, six, and twenty classes.

### Reduced amino acid alphabets

To define reduced amino acid alphabets, we mainly used a hierarchical clustering proposed by Levy and coworkers [30], shown on the right of Fig. 1. It is based on the following similarity measure between two amino acid types *x*, *y*:

Sxy=∑i=120Mx,iMy,i(∑i=120Mx,iMx,i)(∑i=120My,iMy,i).
 MathType@MTEF@5@5@+=feaafiart1ev1aaatCvAUfKttLearuWrP9MDH5MBPbIqV92AaeXatLxBI9gBaebbnrfifHhDYfgasaacH8akY=wiFfYdH8Gipec8Eeeu0xXdbba9frFj0=OqFfea0dXdd9vqai=hGuQ8kuc9pgc9s8qqaq=dirpe0xb9q8qiLsFr0=vr0=vr0dc8meaabaqaciaacaGaaeqabaqabeGadaaakeaacqWGtbWudaWgaaWcbaGaemiEaGNaemyEaKhabeaakiabg2da9maalaaabaWaaabmaeaacqWGnbqtdaWgaaWcbaGaemiEaGNaeiilaWIaemyAaKgabeaakiabd2eannaaBaaaleaacqWG5bqEcqGGSaalcqWGPbqAaeqaaaqaaiabdMgaPjabg2da9iabigdaXaqaaiabikdaYiabicdaWaqdcqGHris5aaGcbaGaeiikaGYaaabmaeaacqWGnbqtdaWgaaWcbaGaemiEaGNaeiilaWIaemyAaKgabeaakiabd2eannaaBaaaleaacqWG4baEcqGGSaalcqWGPbqAaeqaaOGaeiykaKcaleaacqWGPbqAcqGH9aqpcqaIXaqmaeaacqaIYaGmcqaIWaama0GaeyyeIuoakiabcIcaOmaaqadabaGaemyta00aaSbaaSqaaiabdMha5jabcYcaSiabdMgaPbqabaGccqWGnbqtdaWgaaWcbaGaemyEaKNaeiilaWIaemyAaKgabeaaaeaacqWGPbqAcqGH9aqpcqaIXaqmaeaacqaIYaGmcqaIWaama0GaeyyeIuoakiabcMcaPaaacqGGUaGlaaa@6A44@

*M *is the Blosum50 matrix [34], and the sums are over the twenty amino acid types. The clustering method assigns the two amino acid types with the highest similarity to a group. Then, the pair with the next-highest similarity value is considered. If one member of this pair belongs to the first group, the other member is added to that group. If not, the pair is assigned to a new group. This process is repeated until all the amino acid types are divided into a predefined, desired number of groups.

We also performed our own groupings, using the same clustering method, but a different similarity measure, defined by our own empirical energy function. In this case, the similarity between two amino acid types was related to the similarity between the associated coefficients in the energy matrix *U*, constructed with the complete amino acid alphabet of twenty types. The pairwise similarity was defined as the Pearson correlation coefficient between their sets of coefficients in the energy matrix *U*:

Sxy=∑i=120((Ux,i−Ux¯)(Uy,i−Uy¯))∑i=120(Ux,i−Ux¯)2∑j=120(Uy,i−Uy¯)2
 MathType@MTEF@5@5@+=feaafiart1ev1aaatCvAUfKttLearuWrP9MDH5MBPbIqV92AaeXatLxBI9gBaebbnrfifHhDYfgasaacH8akY=wiFfYdH8Gipec8Eeeu0xXdbba9frFj0=OqFfea0dXdd9vqai=hGuQ8kuc9pgc9s8qqaq=dirpe0xb9q8qiLsFr0=vr0=vr0dc8meaabaqaciaacaGaaeqabaqabeGadaaakeaacqWGtbWudaWgaaWcbaGaemiEaGNaemyEaKhabeaakiabg2da9maalaaabaWaaabmaeaacqGGOaakcqGGOaakcqWGvbqvdaWgaaWcbaGaemiEaGNaeiilaWIaemyAaKgabeaakiabgkHiTiabdwfavnaaBaaaleaacuWG4baEgaqeaaqabaGccqGGPaqkcqGGOaakcqWGvbqvdaWgaaWcbaGaemyEaKNaeiilaWIaemyAaKgabeaakiabgkHiTiabdwfavnaaBaaaleaacuWG5bqEgaqeaaqabaGccqGGPaqkcqGGPaqkaSqaaiabdMgaPjabg2da9iabigdaXaqaaiabikdaYiabicdaWaqdcqGHris5aaGcbaWaaabmaeaacqGGOaakcqWGvbqvdaWgaaWcbaGaemiEaGNaeiilaWIaemyAaKgabeaakiabgkHiTiabdwfavnaaBaaaleaacuWG4baEgaqeaaqabaGccqGGPaqkdaahaaWcbeqaaiabikdaYaaakmaaqadabaGaeiikaGIaemyvau1aaSbaaSqaaiabdMha5jabcYcaSiabdMgaPbqabaGccqGHsislcqWGvbqvdaWgaaWcbaGafmyEaKNbaebaaeqaaOGaeiykaKYaaWbaaSqabeaacqaIYaGmaaaabaGaemOAaOMaeyypa0JaeGymaedabaGaeGOmaiJaeGimaadaniabggHiLdaaleaacqWGPbqAcqGH9aqpcqaIXaqmaeaacqaIYaGmcqaIWaama0GaeyyeIuoaaaaaaa@768F@

Here, *x *and *y *are two different amino acids, *U*_*x*, *i *_is the pairwise energy of interaction between the residues *x *and *i*, Ux¯
 MathType@MTEF@5@5@+=feaafiart1ev1aaatCvAUfKttLearuWrP9MDH5MBPbIqV92AaeXatLxBI9gBaebbnrfifHhDYfgasaacH8akY=wiFfYdH8Gipec8Eeeu0xXdbba9frFj0=OqFfea0dXdd9vqai=hGuQ8kuc9pgc9s8qqaq=dirpe0xb9q8qiLsFr0=vr0=vr0dc8meaabaqaciaacaGaaeqabaqabeGadaaakeaacqWGvbqvdaWgaaWcbaGafmiEaGNbaebaaeqaaaaa@2F9C@ is the average over the *U*_*x*, *i*_, and similarly for *y*.

### Optimizing the energy function: general method

We define different energy functions, corresponding to amino acid alphabets of varying complexities. For a given amino acid alphabet, the energy function is optimized by adjusting the elements of the corresponding energy matrix *U*. The goal is to assign low energies to conformations that ressemble the native structure (which can be monomeric or dimeric). We follow a method introduced by Bastolla and coworkers [20]. To characterize the similarity between two conformations, with the contact maps *C *and *C'*, we consider the number *q*(*C*, *C'*) of contacts that are present in both maps, divided by the number of contacts in the larger of the two maps (the one with the most contacts). *q*(*C*, *C'*) is referred to as the "contact similarity". Then; for any native structure, we compute the Boltzmann-averaged overlap *Q *with all of its decoys:

Q=∑Cq(C,Cnat)e−E(C)/kT∑Ce−E(C)/kT,
 MathType@MTEF@5@5@+=feaafiart1ev1aaatCvAUfKttLearuWrP9MDH5MBPbIqV92AaeXatLxBI9gBaebbnrfifHhDYfgasaacH8akY=wiFfYdH8Gipec8Eeeu0xXdbba9frFj0=OqFfea0dXdd9vqai=hGuQ8kuc9pgc9s8qqaq=dirpe0xb9q8qiLsFr0=vr0=vr0dc8meaabaqaciaacaGaaeqabaqabeGadaaakeaacqWGrbqucqGH9aqpdaWcaaqaamaaqababaGaemyCaeNaeiikaGIaem4qamKaeiilaWIaem4qam0aaSbaaSqaaiabd6gaUjabdggaHjabdsha0bqabaGccqGGPaqkcqWGLbqzdaahaaWcbeqaaiabgkHiTiabdweafjabcIcaOiabdoeadjabcMcaPiabc+caViabdUgaRjabdsfaubaaaeaacqWGdbWqaeqaniabggHiLdaakeaadaaeqaqaaiabdwgaLnaaCaaaleqabaGaeyOeI0IaemyrauKaeiikaGIaem4qamKaeiykaKIaei4la8Iaem4AaSMaemivaqfaaaqaaiabdoeadbqab0GaeyyeIuoaaaGccqGGSaalaaa@538F@

where the sum is over all the decoys, *C *is the contact map of a decoy, *C*_*nat *_is the native contact map, *k *is Boltzmann's constant, and *T *is the absolute temperature. If the Boltzmann-averaged overlap *Q *is close to one, then the lowest-energy conformation is either equal to the native structure or very close to it. At physiological temperatures, a high value of *Q *implies that structures very different from the native one have high energies, while native-like structures have low energies. The energy landscape is said to be well-correlated [20].

The interaction parameters are chosen to maximize the quantity Q¯
 MathType@MTEF@5@5@+=feaafiart1ev1aaatCvAUfKttLearuWrP9MDH5MBPbIqV92AaeXatLxBI9gBaebbnrfifHhDYfgasaacH8akY=wiFfYdH8Gipec8Eeeu0xXdbba9frFj0=OqFfea0dXdd9vqai=hGuQ8kuc9pgc9s8qqaq=dirpe0xb9q8qiLsFr0=vr0=vr0dc8meaabaqaciaacaGaaeqabaqabeGadaaakeaacuWGrbqugaqeaaaa@2DEF@,

Q¯(U)=∑SQ(S,U),
 MathType@MTEF@5@5@+=feaafiart1ev1aaatCvAUfKttLearuWrP9MDH5MBPbIqV92AaeXatLxBI9gBaebbnrfifHhDYfgasaacH8akY=wiFfYdH8Gipec8Eeeu0xXdbba9frFj0=OqFfea0dXdd9vqai=hGuQ8kuc9pgc9s8qqaq=dirpe0xb9q8qiLsFr0=vr0=vr0dc8meaabaqaciaacaGaaeqabaqabeGadaaakeaacuWGrbqugaqeaiabcIcaOiabdwfavjabcMcaPiabg2da9maaqafabaGaemyuaeLaeiikaGIaem4uamLaeiilaWIaemyvauLaeiykaKcaleaacqWGtbWuaeqaniabggHiLdGccqGGSaalaaa@3C35@

where the sum is over all the proteins of our Optimization set. Following Bastolla et al, we optimize Q¯
 MathType@MTEF@5@5@+=feaafiart1ev1aaatCvAUfKttLearuWrP9MDH5MBPbIqV92AaeXatLxBI9gBaebbnrfifHhDYfgasaacH8akY=wiFfYdH8Gipec8Eeeu0xXdbba9frFj0=OqFfea0dXdd9vqai=hGuQ8kuc9pgc9s8qqaq=dirpe0xb9q8qiLsFr0=vr0=vr0dc8meaabaqaciaacaGaaeqabaqabeGadaaakeaacuWGrbqugaqeaaaa@2DEF@ through a gradient ascent method, adjusting the energy matrix *U *iteratively, according to the rule:

U(t+1)=U(t)+δ∑S∇U−δγ∑S(1−q0q0)E(Cnat)∇U
 MathType@MTEF@5@5@+=feaafiart1ev1aaatCvAUfKttLearuWrP9MDH5MBPbIqV92AaeXatLxBI9gBaebbnrfifHhDYfgasaacH8akY=wiFfYdH8Gipec8Eeeu0xXdbba9frFj0=OqFfea0dXdd9vqai=hGuQ8kuc9pgc9s8qqaq=dirpe0xb9q8qiLsFr0=vr0=vr0dc8meaabaqaciaacaGaaeqabaqabeGadaaakeaacqWGvbqvdaahaaWcbeqaaiabcIcaOiabdsha0jabgUcaRiabigdaXiabcMcaPaaakiabg2da9iabdwfavnaaCaaaleqabaGaeiikaGIaemiDaqNaeiykaKcaaOGaey4kaSccciGae8hTdq2aaabuaeaacqGHhis0cqWGvbqvcqGHsislcqWF0oazcqWFZoWzaSqaaiabdofatbqab0GaeyyeIuoakmaaqafabaWaaeWaaeaadaWcaaqaaiabigdaXiabgkHiTiabdghaXnaaBaaaleaacqaIWaamaeqaaaGcbaGaemyCae3aaSbaaSqaaiabicdaWaqabaaaaaGccaGLOaGaayzkaaaaleaacqWGtbWuaeqaniabggHiLdGccqWGfbqrcqGGOaakcqWGdbWqdaWgaaWcbaGaemOBa4MaemyyaeMaemiDaqhabeaakiabcMcaPiabgEGirlabdwfavbaa@5C44@

where *t*, *t *+ 1 are successive iterations; the sum is over the proteins *S *in the Optimization set; *q*_0 _is the overlap between the minimum energy conformation and the native structure of *S*; *δ *and *γ *are numerical parameters, chosen to facilitate convergence to a maximum. Typical values were 0.2 and 0.0075. For more details, see [20].

For monomer structure recognition, we optimized the energy function using the 615 native structures in our Optimization set, along with their associated decoys. For dimer recognition, we optimized the energy function using a combination of monomeric and dimeric structures. We considered two Optimization sets, each containing 109 or 110 dimeric structures and their associated decoys, along with 110 monomeric structures and their decoys. We denote the Optimization sets by OS1 and OS2. The dimeric structures were assigned randomly to OS1 or OS2. The monomeric structures were chosen so that their average chain length is equal to that of the Monomeric Optimization Set, above (189 amino acids). The OS2 dimer structures form the Test Set 1 (TSl); the OS1 dimer structures form Test Set 2 (TS2). The same monomeric structures were used for both OS1 and OS2. Notice that cross-validation, below, is done using the performance of the energy functions on interface recognition only. Parameter bias towards the monomer structures can exist but is not a concern. Notice also that with the normalization of *Q *in Eq. (6), the monomers and dimers have the same weight in the combined optimization, despite the larger number of decoys for the dimers.

### Optimizing the energy function: starting parameters

For the more complex alphabets, the number of energy parameters to be optimized is large (210 in the case of the complete amino acid alphabet). This means that there are many local maxima for Q¯
 MathType@MTEF@5@5@+=feaafiart1ev1aaatCvAUfKttLearuWrP9MDH5MBPbIqV92AaeXatLxBI9gBaebbnrfifHhDYfgasaacH8akY=wiFfYdH8Gipec8Eeeu0xXdbba9frFj0=OqFfea0dXdd9vqai=hGuQ8kuc9pgc9s8qqaq=dirpe0xb9q8qiLsFr0=vr0=vr0dc8meaabaqaciaacaGaaeqabaqabeGadaaakeaacuWGrbqugaqeaaaa@2DEF@, and the optimization must be done repeatedly, with multiple starting points. We used three methods, both for the monomeric and dimeric protein sets. For the simplest alphabets, with two or three amino acid types, we performed a systematic scan of possible parameter choices. The range of values considered was from -10 to + 10 kcal/mol, with a step of 0.25 (binary alphabet) or 0.5 (ternary alphabet). For the larger alphabets, a systematic scan was no longer possible; instead, we used 64000 random starting points, with each parameter drawn randomly from the same range of -10 to +10 kcal/mol. In addition, for alphabets with three amino acid types or more, we used starting points taken from the optimization of the smaller alphabets. Indeed, the alphabets form a hierarchy, where the finer groupings are subdivisions of the larger groupings. For example, the binary alphabet uses the groups {LVIMCAGSTPFYW} (hydrophobic) and {EDNQKRH} (hydrophilic), while the ternary alphabet splits the hydrophobic group into two subgroups.

The ternary optimization can be started from the binary optimum, assigning initially the same, "binary" parameters to the two hydrophobia subgroups. As the optimization proceeds, the parameters associated with two subgroups will shift to different values.

For the alphabets with 6 and 20 classes, to explore the effect of an overall scaling of the energy parameters, we also generated sets of random starting parameters with a fine-grained sampling of possible values, limited to the range -1 to +1 kcal/mol. These parameter sets were optimized in combination with four different values of the temperature *T *(Eq. 6), corresponding to *kT *products of 0.6, 1.0, 1.5, and 4.0 kcal/mol. 2000 parameter sets were optimized with each temperature, for each each alphabet, giving 8000 additional parameter sets per alphabet.

### Testing the energy function

To test the potentials, we used a cross validation procedure. As described above, the set of native structures were split into Optimization and Test sets. Only the Optimization set was used in Eqs. (7, 8), above. The total Q¯
 MathType@MTEF@5@5@+=feaafiart1ev1aaatCvAUfKttLearuWrP9MDH5MBPbIqV92AaeXatLxBI9gBaebbnrfifHhDYfgasaacH8akY=wiFfYdH8Gipec8Eeeu0xXdbba9frFj0=OqFfea0dXdd9vqai=hGuQ8kuc9pgc9s8qqaq=dirpe0xb9q8qiLsFr0=vr0=vr0dc8meaabaqaciaacaGaaeqabaqabeGadaaakeaacuWGrbqugaqeaaaa@2DEF@ was then evaluated for the Test set. A value close to 100% means that the native structure is almost always ranked as the lowest-energy conformation. We also compute a "discrimination" percentage. We mostly employ a "strong" discrimination, *D*, which is the fraction of native structures that are ranked as the lowest-energy conformation (compared to their decoy set). We occasionally compute a "weaker" discrimination, *D*_*k *_, which is the fraction of native structures that are ranked among the *k *lowest-energy conformations (so that *D *= *D*_1_).

Another form of cross validation was done by performing several blind tests. In one test, we used our energy functions to rank native and decoy structures available on the web, from Vakser, Sternberg and coworkers. Vakser's decoys sets were produced by a rigid body docking method, using the GRAMM program at "high resolution" (grid step ≤ 2 Å) [27]. They correspond to five biological dimeric complexes. Each decoy series includes 99 decoys. A few of these are within 5 Å (rms deviation) of the native structure; the others are farther away. The Sternberg decoys were produced by a rigid body docking method using the MultiDock program [28]. They correspond to ten biological dimeric complexes, including one that is present in the Vakser set (with different decoys). Each series includes 99 decoys. An electrostatic filter is used during the docking simulations, and all ten decoy series include at least three decoys that are close to the native structure. The complexes are listed in Supplementary Material.

Finally, in another blind test, we applied our energy functions to native and decoy structures of protein dimers submitted to the CAPRI experiment for protein–protein complex structure prediction (rounds 2–5) [8,9]. We considered 13 native structures, with an average of 173 decoys each and a minimum of 66.

## Authors' contributions

GL: Performed calculations, analyzed data, wrote paper. RM: analyzed data. SW: designed research, analyzed data. TS: designed research, analyzed data, wrote paper.

## Supplementary Material

Additional file 1 Convergence and robustness of the energy functions. Some additional data on the energy function refinement, both for fold recognition and interface recognition (text and one table). Includes best six-class energy functions optimized using Optimization Sets 1 and 2. Click here for file

Additional file 2 OS2 20-class energy parameters. A table containing the complete OS2 20-class energy matrix (kcal/mol). Click here for file

Additional file 3 Biological complexes for blind tests. List of biological complexes used for the Sternberg and Vakser blind tests, including information on the hydrophobic/hydrophilic nature of the interfaces. Click here for file
